# Site-specific Isopeptide Bridge Tethering of Chimeric gp41 N-terminal Heptad Repeat Helical Trimers for the Treatment of HIV-1 Infection

**DOI:** 10.1038/srep32161

**Published:** 2016-08-26

**Authors:** Chao Wang, Xue Li, Fei Yu, Lu Lu, Xifeng Jiang, Xiaoyu Xu, Huixin Wang, Wenqing Lai, Tianhong Zhang, Zhenqing Zhang, Ling Ye, Shibo Jiang, Keliang Liu

**Affiliations:** 1 State Key Laboratory of Toxicology and Medical Countermeasures, Beijing Institute of Pharmacology & Toxicology, 27 Tai-Ping Road, Beijing, 100850, China; 2 Key Laboratory of Medical Molecular Virology of Ministries of Education and Health, School of Basic Medical Sciences, Fudan University, Shanghai 200032, China; 3 School of Pharmaceutical Engineering, Shenyang Pharmaceutical University, Shenyang, 110016, China; 4 Department of Microbiology and Immunology and Emory Vaccine Center, Emory University School of Medicine, Atlanta, GA 30322, USA; 5 Lindsley F. Kimball Research Institute, New York Blood Center, New York, NY 10065, USA

## Abstract

Peptides derived from the N-terminal heptad repeat (NHR) of HIV-1 gp41 can be potent inhibitors against viral entry when presented in a nonaggregating trimeric coiled-coil conformation via the introduction of exogenous trimerization motifs and intermolecular disulfide bonds. We recently discovered that crosslinking isopeptide bridges within the *de novo* helical trimers added exceptional resistance to unfolding. Herein, we attempted to optimize (CCIZN17)_3_, a representative disulfide bond-stabilized chimeric NHR-trimer, by incorporating site-specific interhelical isopeptide bonds as the redox-sensitive disulfide surrogate. In this process, we systematically examined the effect of isopeptide bond position and molecular sizes of auxiliary trimeric coiled-coil motif and NHR fragments on the antiviral potency of these NHR-trimers. Pleasingly, (IZ14N24N)_3_ possessed promising inhibitory activity against HIV-1 infection and markedly increased proteolytic stability relative to its disulfide-tethered counterpart, suggesting good potential for further development as an effective antiviral agent for treatment of HIV-1 infection.

The HIV-1 envelope glycoprotein (Env) gp120/gp41 complex promotes viral infection by mediating the fusion between viral and cell membranes^1^. Upon gp120 binding to the cellular receptor CD4, along with a coreceptor, a series of conformational changes occur in gp41^2^, culminating in the formation of a fusogenic six-helix bundle (6HB) via the gp41 C-terminal heptad repeat (CHR) and the central N-terminal heptad repeat (NHR) trimer interaction that pulls viral and cellular membranes together for fusion^3^,^4^. Peptides derived from NHR or CHR sequences, designated as N- or C-peptides, respectively, can abrogate the virus-cell fusion process by competitively blocking the fusogenic 6HB formation (Fig. 1a)^5^. One of the C-peptides, T20 (Enfuvirtide, Fuzeon), is the first and only U.S. Federal Drug Administration-approved HIV-1 fusion inhibitor^6^. Because of the rapid appearance of drug-resistant HIV-1 strains and the short *in vivo* half-life of T20, it is critical to develop new generations of fusion inhibitors to allow for the very likely contingency of resistance development and to obtain higher proteolytic stability^7^,^8^.

In contrast to C-peptides with low nanomolar antiviral activity, N-peptides can only inhibit HIV-1 entry at micromolar concentrations^9^. One barrier against the development of potent N-peptides involves the strong aggregation properties of synthetic NHR-based peptides when taken out of their parent protein surroundings^10^,^11^. Therefore, researchers have long sought chemical interventions to recapitulate the bioactive trimeric coiled-coil structure of these N-peptides^12^,^13^. Different design approaches have addressed the necessity of stabilizing the helical trimer conformation of N-peptides. One that deserves particular attention involves the construction of chimeric molecules incorporating the attachment of an exogenous solubilizing trimerized motif to an NHR peptide and further covalent stabilization of these trimers via disulfide bonds^13^,^14^. As eminent examples of covalent chimeric constructs, (CCIZN17)_3_ and ccN28Fd fold as highly stable helical trimers that exhibit strong antifusion potency against various HIV-1 isolates, including those resistant to clinically used T20. Despite these advances, such exogenous trimerization motifs usually inherit a lengthy sequence, such as IQ (28 residues), IZ (24 residues), and Fd (27 residues)^12^,^15^. These extra-large trimerization motifs may be detrimental to the ability of these inhibitors to target the gp41 CHR region, thus attenuating their antiviral activity. Furthermore, possible alterations in the disulfide structure resulting from disulfide isomerases and thiols *in vivo*^16^,^17^, as well as the varied redox properties in the biological milieu maintained by the surrounding cell populations and organs, may result in the loss of activity of these chimeric molecules intended for therapeutic use^18^,^19^.

Ultra-high stability provided by isopeptide bond crosslinks is present in a range of bacterial surface proteins. Inspired by this, we very recently developed an isopeptide bridge-tethering strategy for the stabilization of *de novo* coiled coils with simple peptide sequences^20^. In the present work, we performed lead optimization based on the scaffold of (CCIZN17)_3_ in which isopeptide bonds were incorporated into the IZ motif, a well-folded trimeric coiled coil with an extremely complex chemical environment, to replace the interhelical disulfide at the N-terminus of the chimeric peptide. In the process of developing these isopeptide bond-tethered NHR-trimer mimetics, we examined the site-specificity for the isopeptide bridge insertion and performed a detailed study to identify the optimal combination of isopeptide bond position, IZ motif truncation, and N-peptide length in the chimeric molecules. One of these isopeptide bridge-tethered chimeric peptides, (IZ14N24N)_3_, containing only half as many residues in the exogenous trimerization motif as (CCIZN17)_3_ and displaying low nanomolar activity against HIV-1 fusion, was further subjected to an *in vitro* metabolic stability assay and compared with its disulfide-tethered counterpart. This study lays the foundation for further optimization of these coiled-coil mimetics as potent and metabolically stable inhibitors against HIV-1. It is expected that these efforts could be useful in the rational design of stable helical trimers, interfering with coiled-coil 6HB formation present in other enveloped viruses with class I fusion proteins.

## Results and Discussion

### Design

**Figure 1:**
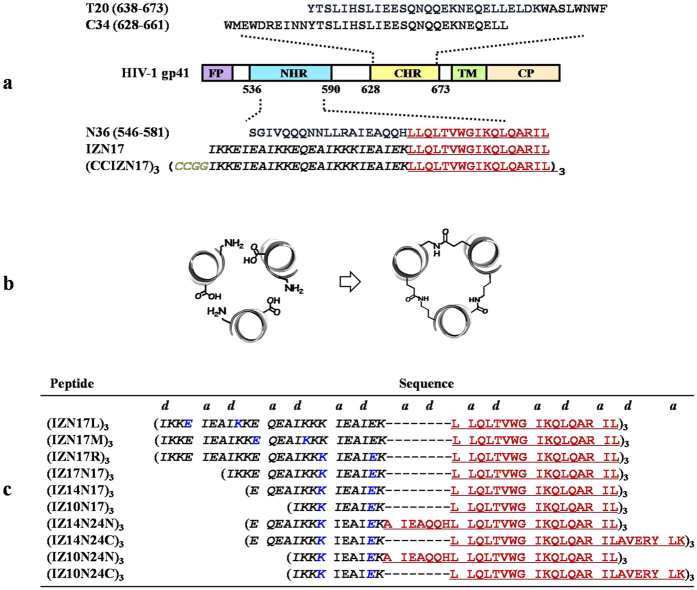
Schematic representation of HIV-1 gp41, cartoon representation of the isopeptide bond-tethered trimeric coiled coil, and the designed chimeric N-peptides. (**a**) The HIV-1 gp41 functional domains. FP, fusion peptide; NHR, N-terminal heptad repeat; CHR, C-terminal heptad repeat; TM, transmembrane domain; CP, cytoplasmic domain. The representative C-peptides, i.e., T20 and C34, and chimeric NHR-trimers, i.e., IZN17 and (CCIZN17)_3_, are shown in the diagram. (**b**) Use of isopeptide bridges in place of interstrand ionic interactions at the *g-e’* positions. (**c**) Peptide sequences of our designed (CCIZN17)_3_ derivatives. The specific Lys-Glu isopeptide bonds are shown in blue. The NHR sequences are highlighted in red and underlined. The N-terminus and C-terminus of each peptide were acetylated and amidated, respectively.

To avoid the aggregation of linear N-peptides in physiological solutions, Kim’s group has designed the chimeric molecule IZN17 in which the primary pocket-forming sequence in the gp41 NHR region, i.e., N17, is fused to the C-terminus of a designed trimeric coiled-coil motif, i.e., IZm (IZ)^15^. Moreover, introducing a CCGG motif to the N-terminus of IZN17 resulted in the formation of intermolecular disulfide bridges between the subunits of the IZN17 trimer. This produced an NHR trimer inhibitor with even more potency: (CCIZN17)_3_ (Fig. 1a)^14^. Therefore, in this study, (CCIZN17)_3_ was selected as the lead compound, and chimeric peptides with a smaller auxiliary motif, higher antiviral activity, and improved proteolytic stability were subsequently constructed. The IZ sequence incorporates favorable intrastrand ionic interactions as well as three pairs of judiciously placed interhelical ionic *i* to *i*′ + 5 (*g-e*′) interactions to enhance both the helical and the coiled-coil stability. Our seminal work, which investigates the stabilities of *de novo* helical trimers with simple peptide sequences, offers a promising approach for stabilizing coiled coils by placing isopeptide linkages in place of interhelical electrostatic contacts (Fig. 1b). We postulated that the isopeptide bridge-tethering strategy would also be applicable for the IZ motif to substitute the disulfide bonds in (CCIZN17)_3_. Therefore, we designed the first series of chimeric trimers, i.e., (IZN17L)_3_, (IZN17M)_3_, and (IZN17R)_3_. In these chimeric trimers, isopeptide bridges are placed at different *g-e’* positions within IZN17 such that they are incorporated between the Glu-4 and Lys-9 side chains, between the Glu-11 and Lys-16 side chains, and between the Lys-18 and Glu-23 side chains, respectively (Fig. 1c). Furthermore, to minimize the size of the exogenous trimerization motif, we designed a second series of truncated chimeric peptides, including (IZ17N17)_3_, (IZ14N17)_3_, and (IZ10N17)_3_, with a 7-, 10-, and 14-amino acid deletion from the N-terminus of each subunit of (IZN17R)_3_, respectively. Lastly, the influence of additional functional domains in the viral gp41 NHR region on the antiviral activity of the chimeric peptides was explored. To do this, N24N peptide, containing both the primary pocket domain and its contiguously upstream helix zone domain-derived 7-mer peptide, and N24C peptide, containing both the primary pocket and its downstream ^579^RILAVERYLK^588^ motif-based subpocket^21^, were fused to the C-terminus of the IZ14-motif and IZ10-motif, respectively, to construct the third series of chimeric N-peptides (Fig. 1c).

### Site-specific isopeptide bridge insertion in helical trimers with an extremely complex chemical environment

**Figure 2:**
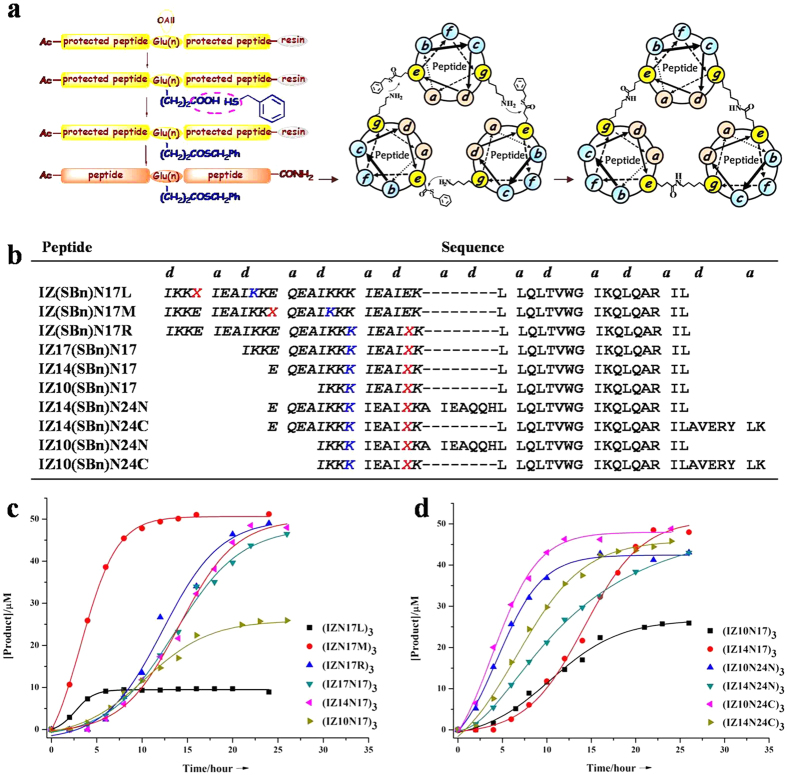
Reactions schemes, sequences of thioester-modified peptides, and the product formation versus time for acyl transfer reaction. (**a**) Schematic representation of the strategy used to prepare crosslinked chimeric N-peptides. The N in parentheses represents the positions of the active-site Glu residues modified with a thioester as an acyl donor. Helical wheel diagram of the trimeric coiled-coils illustrates the three symmetry-related active sites juxtaposing an acyl-acceptor and -donor moieties at *i* to *i*′ + *5* positions. (**b**) Peptide sequences of the thioester intermediates. Glu residues with a thioester side chain, designated as X, and active site Lys residues are highlighted in red and blue, respectively. (**c,d**) Acyl transfer product formation in time initiated with 150 μM thioester-peptide precursor in PBS/H_2_O/CH_3_CN (3:5:2, v/v).

To synthesize the isopeptide bridge-tethered chimeric N-peptides, an interhelical acyl-transfer reaction was used (Fig. 2a). It relied on thioester-modified trimeric coiled-coil assemblies to juxtapose the acyl donor and acceptor moieties as competent active sites^20^,^22^. In general, a Glu residue with an allyloxy group (OAll)-modified side chain was placed at the engineered active-site positions within the IZN17 peptide and its derivatives to substitute the original Glu residues. After standard solid-phase Fmoc synthesis, the selective deprotection of the OAll group was achieved on-resin via palladium-catalyzed deprotection. Then, a benzyl thioester (SBn) was introduced to the side chain of the Glu residue, followed by regular TFA-mediated cleavage and purification. Thereafter, the thioester-modified peptides were used as intermediates to generate the cross-linked gp41 N-trimer mimetics (Fig. 2b), and the reaction was monitored by reversed-phase HPLC. As shown in Fig. 2c, the precursor IZ(SBn)N17L exhibited rapid substrate hydrolysis, suggesting that the acyl-donor moiety placed near the peptide N-terminus markedly decreased the transfer efficiency. It should be noted that truncation of the 24-residue IZ sequence to a 14-residue exogenous peptide had little effect on the reaction. However, IZ10(SBn)N17 displayed a dramatically reduced transfer rate, combined with competing thioester hydrolysis. Encouragingly, extending the N-peptide length restored the efficiency of the reaction in sequences with a short IZ motif and resulted in acyl transfer with a rate higher than that for their N17 counterparts. Moreover, the reduced transfer rates for IZ14(SBn)N24N and IZ14(SBn)N24C, compared to those for IZ10(SBn)N24N and IZ10(SBn)N24C, most likely resulted from the increased steric hindrance near the acyl acceptor moieties (Fig. 2d). To probe the specificity of the interhelical acyl-transfer reaction that occurred in the IZ motif, we disabled the active-site acyl-donor residues in each peptide intermediate using an Arg residue (Supplementary Table S1). No reaction was observed in these control experiments, suggesting that our previously developed isopeptide bridge-tethering strategy could be extended to the site-directed crosslinking of superhelical scaffolds with a more challenging chemical environment (Supplementary Fig. S1).

### Exceptional resistance of covalently stabilized N-trimers to thermal denaturation

**Figure 3:**
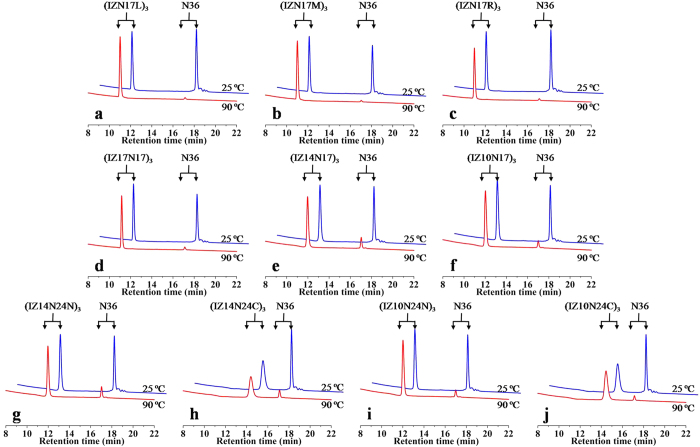
Thermal tolerance of isopeptide bridge-tethered chimeric N-trimers. A mixture of each crosslinked chimeric N-trimer (100 μM) and N36 peptide (300 μM) in 100 mM PBS (pH 7.2) was heated at the indicated temperature for 10 min and then centrifuged, followed by analysis of the supernatant by reversed-phase HPLC.

To assess the nature and conformational stability of the crosslinked N-trimers, CD spectroscopy was used. Data from the CD analysis indicated that all chimeric N-trimers formed typical α-helices, characterized by a double minima at 208/222 nm, with α-helicity ranging from 75% to 100% (Supplementary Table S2). Impressively, these trimeric helical scaffolds showed high thermal stability with minimal denaturation and no cooperative melting transition at temperatures up to 90 °C at 10 μM concentrations. To further test the thermal stability of the isopeptide bridge-tethered chimeric helical trimers, we incubated mixtures of a chimera and N36, a representative free N-peptide prone to aggregation under physiological conditions, at 25 °C or 90 °C. It was conceivable that such mixing could eliminate interference originating from different experimental conditions. Notably, it was found that the crosslinked chimeras did not aggregate in solution, even up to 90 °C, whereas free N36 peptide completely aggregated (Fig. 3).

### Isopeptide bridge-tethered chimeric N-peptides exhibited highly potent anti-HIV-1 activities

We determined the ability of these chimeric N-peptides to interfere with the fusion process in an HIV-1 Env-mediated cell-cell fusion assay (Table 1). In our assay, (IZN17L)_3_, (IZN17M)_3_, and (IZN17R)_3_ had EC_50_ values of 20.2, 13.9, and 10.7 nM, respectively, similar to that of (CCIZN17)_3_. These results indicate that isopeptide bridges can be introduced into proteins as a disulfide bond surrogate and that their position of incorporation has little effect on the activity of the chimeric peptides against cell fusion. Subsequently, we sought to determine whether IZ motif truncation would impact their inhibitory activity. It was evident that (IZ17N17)_3_ and (IZ14N17)_3_ continued to maintain potent cell-cell fusion inhibitory activity. Unfortunately, further truncation could not be tolerated, and (IZ10N17)_3_ exhibited 17-fold less potency than (IZN17R)_3_. These findings suggest that the appropriate size of the exogenous trimerization motif contributes significantly to the anti-HIV-1 activity of the trimeric N-peptide fusion inhibitors. Strikingly, attaching N24N to the C-terminus of the shortened IZ14- or IZ10-motif dramatically increased the activity. One of them, (IZ14N24N)_3_, had an EC_50_ value of 2.79 nM, which was about 8-fold greater than that of (IZ14N17)_3_, reaching that of T20 and proving more potent than (CCIZN17)_3_. Similarly, the enhanced potency caused by the extended gp41 NHR peptide was also observed in N24C-fused NHR trimers. In contrast to the binding of small molecules to well-defined clefts or cavities in enzymes, the interactions between peptides and ‘hot segments’ in a relatively flat and featureless protein-protein interface are characterized by much more shallow interactions that can be widely distributed over the interfacial areas. Compared with N17 peptide, N24N and N24C, which possess more functional domains, span a larger portion of the protein surface and enable many more favorable interactions, thus generating more effective antiviral activity. We and others have shown previously that extending the length of the gp41 NHR segment in the chimeric N-peptides with extra-large trimerized auxiliary motifs, e.g., IZ (24 residues) and Fd (27 residues), results in their decreased inhibitory activity^12^,^14^. We speculate that the lengthy trimerized auxiliary peptides combined with extended N-peptides may lead to bulky chimeric molecules that can be detrimental to access to the gp41 CHR domain, thus attenuating their antiviral activity. However, the isopeptide bond insertion in a downsized auxiliary motif would aid in the construction of chimeric N-peptide inhibitors with an appropriate molecular size in the process of extending the effective N-peptide length, thus leading to a positive correlation between the length of the NHR segment and the inhibitory activity of the chimeras.

Next, the inhibitory activities of the compounds were tested in viral replication assays using laboratory-adapted HIV-1 IIIB (subtypes B, X4) and HIV-1 BaL (subtypes B, R5). As shown in Table 1, a strong correlation was observed between inhibition of HIV-1 Env-mediated cell-cell fusion and inhibition of HIV-1 infection. (IZ14N17)_3_, with half of the residues deleted from the exogenous trimerization motif of (CCIZN17)_3_, inhibited HIV-1 infection at a comparable level. In line with its moderate α-helicity, (IZ10N17)_3_ exhibited a dramatic loss of antiviral potency. The most potent compound in the cell-cell fusion assay, i.e., (IZ14N24N)_3_, also strongly inhibited HIV-1_IIIB_ and HIV-1_BaL_ infection with EC_50_ values of 0.4 and 3.2 nM, respectively, and was more potent than T20. Furthermore, all isopeptide bond-tethered trimeric N-peptides had low or absent cytotoxicity, and the most active compound, (IZ14N24N)_3_, had a high selectivity index value of greater than 50,000 (Table 1).

Interestingly, the monomeric C-peptide T-20 was more potent than most of the trimeric N-peptides in inhibiting HIV-1 Env-mediated cell-cell fusion, while T-20 was less effective than most of these trimeric N-peptides for inhibiting virus-cell infection. We believe that this may be attributed to the difference of the target spatial accessibility in these two assays. The HIV-1 Env on the effector cell located in the space between the effector and target cells during the Env-mediated cell-cell fusion is less accessible to the trimeric N-peptide than the monomeric C-peptide T-20 with a molecular size about one third of the trimeric N-peptide. However, both the monomic C-peptide and trimeric N-peptide may have similar accessibility to the HIV-1 Env on a virion located in the space between the virus and target cells during virus-cell infection.

### Isopeptide bond-stabilized chimeric N-trimers are highly potent against HIV-1 strains resistant to T20

T20 is the only fusion inhibitor utilized in treating HIV/AIDS patients who did not respond to conventional drug therapy^23^,^24^. Due to the rapid emergence of T20-resistant HIV-1 variants, new-generation fusion inhibitors are highly desirable^25^,^26^. Based on their completely different structure and binding target compared with those of C-peptides, the development of N-peptide fusion inhibitors will be a new strategy to avoid cross-drug resistance with T20. Therefore, we tested the most active compound (IZ14N24N)_3_ against HIV-1 strains resistant to T20. As shown in Table 2, T20 was effective against the T20-sensitive strain HIV-1_NL4-3(D36G)_, but it was more than 8-fold less effective against T20-resistant strains. However, (IZ14N24N)_3_ showed almost equal potency against both T20-sensitive and -resistant strains, with EC_50_ values of 0.7–3.3 nM. Therefore, although we cannot exclude the possibility that the HIV-1 variants with mutations in the gp41 CHR region may escape from the chimeric N-peptide inhibition, (IZ14N24N)_3_ represents a promising advancement in anti-HIV microbicide design for treating patients refractory to current T20 therapy.

### Dramatically increased resistance of (IZ14N24N)_3_ to proteolysis compared to its disulfide bond-stabilized counterpart

**Figure 4:**
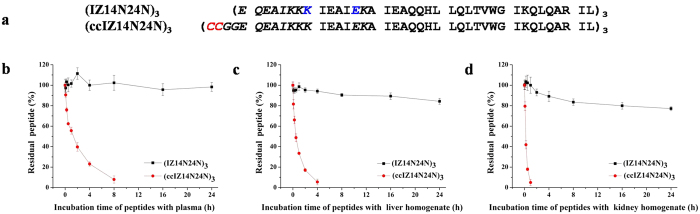
Metabolic stability of the isopeptide bridge-tethered chimeric peptide (IZ14N24N)_3_ and its disulfide bridge-tethered counterpart (ccIZ14N24N)_3_. (**a**) The sequences of (IZ14N24N)_3_ and (ccIZ14N24N)_3_. The isopeptide-bridging residues in (IZ14N24N)_3_ are shown in blue. The two cysteines introduced in order to form intermolecular disulfide bonds between the three trimer subunits are highlighted in red. Proteolytic stability of (IZ14N24N)_3_ and (ccIZ14N24N)_3_ (20 μM) in (**b**) rat plasma, (**c**) liver homogenate, and (**d**) kidney homogenate.

Protein engineering studies have shown that the introduction of disulfide bridges contributes significantly to coiled-coil chain alignment as well as the general stability of coiled-coil assemblies^27^,^28^. Undoubtedly, the incorporation of intermolecular disulfide bonds offers an attractive option for stabilizing chimeric NHR trimers, thus producing N-peptides with nanomolar antiviral potency. However, extracellular thiol-containing molecules, e.g., cysteine, glutathione, and serum albumin^17^,^19^, together with thiol-protein-disulfide oxido-reductases^16^, have the potential to catalyze disulfide destabilization, resulting in the inactivation of these disulfide bond-stabilized chimeric N-peptides by the loss of the superhelical structure. To test whether crosslinking isopeptide bonds within the subunits of the helical trimers provided significantly increased proteolytic stability compared with that of disulfide bonds, we generated (ccIZ14N24N)_3_, in which the interhelical disulfide linkage for replacing the isopeptide bridge within (IZ14N24N)_3_ was positioned at the N-terminus of the IZ14N24N-based helical trimer (Fig. 4a), and compared the metabolic stability of both modifications within the same peptide in rat plasma as well as in liver and kidney homogenates using reversed-phase HPLC. As shown in Fig. 4b,c, (IZ14N24N)_3_ remained essentially intact after a 24-h incubation in plasma. In sharp contrast, its disulfide bridge-tethered counterpart, (ccIZ14N24N)_3_, showed significantly increased susceptibility to proteolysis and could not be detected after an 8-h incubation. Similarly, (IZ14N24N)_3_ was highly stable in the presence of liver or kidney homogenate for 24 h, whereas (ccIZ14N24N)_3_ was completely degraded in the liver homogenate and kidney homogenate after a 4-h and 1-h incubation, respectively. Taken together, these results suggest that isopeptide bond tethering represents a successful complementary strategy for stabilizing coiled-coil mimetics intended for therapeutic use and outperforms disulfide bond crosslinking in terms of proteolytic stability.

### Binding of isopeptide bridge-stabilized chimeric N-trimers to the HIV-1 gp41 CHR domain

**Figure 5:**
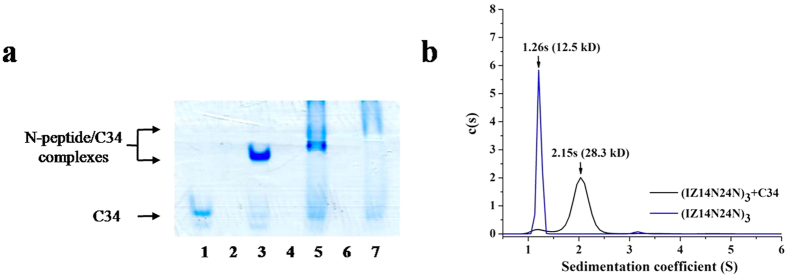
Determination of specific interactions between the N-peptides and C34. (**a**) N-PAGE analysis of N-peptides, C34, and their complexes. Lane 1: C34, lane 2: N36, lane 3: C34 + N36, lane 4: (IZ14N24N)_3_, lane 5: (IZ14N24N)_3_ + C34, lane 6: (IZ10N17)_3_, lane 7: (IZ10N17)_3_ + C34. (**b**) SVA of (IZ14N24N)_3_ and (IZ14N24N)_3_/C34 mixture.

N-peptides interact with gp41 CHR, thereby preventing fusogenic 6HB formation^29^,^30^. CD spectroscopy was first performed to study the interaction between the covalently stabilized N-trimers and their native CHR ligand^31^,^32^. Based on the intensity of the minimum at 222 nm and the 222/208 nm ratio, we found that an equimolar mixture (10 μM) of chimeric N-trimers and C34 provided typical α-helices, with an α-helicity content in the range of 63–87% (Supplementary Table S2). Moreover, a significant difference between the CD spectra of the N-trimers and C34 mixture (Spec_N+C_) and the mathematical sum of the individual spectra of the N- and C-peptides (Spec_N_ + Spec_C_) was observed. These results indicated an interaction between the N-trimers and C34, resulting in enhanced α-helical tertiary structures of the components (Supplementary Fig. S2). As a positive control, the interaction between N36 and C34 led to their dramatic secondary structural changes^4^. In contrast, the mixing of T20 and C34 showed no such structural change, indicating that this peptide pair did not interact^31^. The specific interaction between the most effective compound, (IZ14N24N)_3_, and the HIV-1 gp41 CHR was further confirmed by native (N)-PAGE. As shown in Fig. 5a, C34 showed a band in the lower portion of the gel, while the peptide N36 migrated off the gel because it carries net positive charges under the native electrophoresis conditions. These findings are consistent with a previous study^33^. The mixture of N36 and C34 peptide showed a band corresponding to the 6-HB (N36/C34 complex) in the upper portion of the gel, concomitant with a less intense C34 band. Similarly, (IZ14N24N)_3_ could also form a stable complex with C34 as evidenced by the band in the upper portion of the gel and reduced intensity of the C34 band. However, the (IZ10N17)_3_/C34 mixture displayed two bands with nearly the same density. Based on the intensities of the heterogeneous 6HB bands, the N-PAGE analysis results indicated that (IZ14N24N)_3_ has a stronger interaction with C34 than (IZ10N17)_3_, These findings are consistent with their antiviral activities (Table 1). In addition, the isopeptide bonds cross the grooves of the N-helix trimer, which may slightly perturb the folding of the N-peptides and C34 and lead to imperfect coiled-coil structures, thus appearing as smeared bands in N-PAGE. The size of the complex formed between (IZ14N24N)_3_ and the C34 peptide was further assessed by sedimentation velocity analysis (SVA), which is particularly useful for quantitatively characterizing the behavior of biomolecules in solution^20^. As shown in Fig. 5b, the heterogeneous 6HB states of the isopeptide bridge-tethered chimeric N-peptide (IZ14N24N)_3_ with the C34 peptide were ascertained. Meanwhile, the molecular weights of (IZ14N24N)_3_ was consistent with a trimeric species. Together, these studies indicated that the isopeptide bond-stabilized N-trimers, mimicking the prehairpin intermediate of HIV-1 gp41, are able to bind to the gp41 CHR peptide, C34, to form heterogeneous 6HB complexes, thus inhibiting viral entry and infection.

Jiang’s group and others have made great efforts to identify nonpeptide small-molecule HIV fusion inhibitors that target the highly conserved hydrophobic deep pocket of the gp41 N-trimer; but so far, none of the identified lead compounds possesses anti-HIV-1 potency high enough to enter clinical development^34^,^35^. The lack of an effective high-throughput screening system based on the soluble and stable NHR-trimer with the exposed hydrophobic deep pocket significantly hinders the discovery of highly potent small-molecule HIV-1 fusion inhibitors. One of the fused N-peptide chimeras, IQN17, was proposed to serve as a target for the screening of small molecule HIV fusion inhibitors^36^. However, it is worth noting that IQN17 only presents the primary pocket-forming region of the N-helix. Recently, a subpocket situated at a site downstream of the primary pocket was identified and considered a novel hotspot for small molecule drug design^21^. In our study, the resulting protein (IZ14N24N)_3_, a gp41 NHR-trimer mimetic with shortened exogenous trimerization motifs and an elongated gp41 NHR sequence is remarkably similar to the prehairpin intermediate of gp41. Therefore, (IZ14N24N)_3_ can be used for the development of a high-throughput screening assay for the identification of small-molecule HIV-1 fusion inhibitors and for co-crystallographic analysis of the small-molecule fusion inhibitor/N-trimer complex. Furthermore, it could also be a suitable target for understanding the prefusogenic state of HIV-1 gp41.

## Conclusions

In conclusion, chimeric NHR trimers with anti-HIV-1 fusion activity at low nanomolar levels were designed and synthesized. This was achieved by combining the concepts of grafting an elongated gp41 NHR sequence onto a shortened exogenous IZ motif, with stabilization of the trimeric coiled coils via isopeptide bridges. Our data also showed that isopeptide bridge-tethering could be widely used for stabilizing coiled coil-mediated interactions based on the highly specific interhelical acyl-transfer reaction. This represents an alternative tertiary structure stabilization strategy to the widely used disulfide bridges to afford coiled-coil mimetics with improved metabolic stabilities. Given the common fusogenic mechanism shared by class I viral fusion proteins, we predict that the isopeptide bridge-tethering strategy described herein could be extendable to design NHR-trimer mimetics as microbicides to prevent the transmission of other class I enveloped viruses. Furthermore, this coiled-coil stabilization methodology may also be used to yield new classes of modulators for certain protein-protein interactions (PPIs) owing to the high occurrence of helical bundles at PPI interfaces.

## Methods

### Peptide synthesis

Peptides were synthesized on Rink Amide resin (0.44 mmol/g, Tianjin Nankai Hecheng Sci. & Tech. Co. Ltd.) by using a CEM Liberty peptide synthesizer following the general procedure for Fmoc chemistry of solid-phase peptide synthesis as described previously^20^. Briefly, 0.45 M *O*-benzotriazol-1-yl-*N,N,N’,N’*-tetramethyl-uronium hexafluorophosphate (HBTU, GL Biochem (Shanghai) Ltd.) and 2 M diisopropylethylamine (DIEA, Acrose) in DMF solution were used as coupling reagents. Deprotection was performed using 20% piperidine/DMF. The peptides were cleaved from the resin using TFA/thioanisole/*m*-cresol/water/ethanedithiol in a 82.5:5:5:2.5 volume ratio (Reagent K). The C-terminus of the peptides was amidated, and their N-terminus was acetylated. For peptides with the side chain thioester, Fmoc-Glu(OAll)-OH was used in the thioester-modified site. The allyloxy group was removed by Reagent M, containing tetrakis(triphenylphosphine)palladium(0)/5,5-dimethyl-1,3-cyclohexanedione (1:10 equiv.) in degassed DCM/THF (1:1 v/v, 2 mL) solution. Then the resin was washed five times with 0.5% DIEA in DMF, and five times with 1M sodium diethyldithiocarbamate in DMF. DCC (1 equiv.), 1-hydroxybenzotriazole (HOBt) (1.5 equiv.), and benzyl mercaptan (4 equiv.) were added to the resin for thioester formation. Finally, the resin was cleaved from the resin by Reagent K. For isopeptide bond formation, the precursor thioester-peptide was dissolved at a concentration of 150 μM in PBS/H_2_O/CH_3_CN (3:2:5 v/v), stirred at room temperature for 24 h, and monitored by analytical reversed-phase HPLC. All peptide products were purified by reversed-phase HPLC to >95% purity (Supplementary Fig. S3) and characterized by MALDI-TOF-MS (Autoflex III, Bruker Daltonics Inc., Billerica, MA) (Supplementary Fig. S4).

### Cell-cell fusion assay

The inhibitory activities of these peptides on HIV-1 Env-mediated cell-cell fusion were determined as described previously^37^. Effector cells were HL2/3 cells, which stably express HIV Gag, Env, Tat, Rev, and Nef proteins at the cell surface (contributed by Drs. Barbara Felber and George Pavlakis). TZM-bl cells, which express high levels of CD4 and coreceptors, were used as target cells (contributed by Drs. John C. Kappes and Xiaoyun Wu). HL2/3 cells and TZM-b1 cells were obtained from the NIH AIDS Reference and Reagent Program. TZM-bl cells (2.5 × 10^4^/well) were plated in a 96-well plate (Corning Costar) and cultured overnight at 37 °C in 5% CO_2_. Thereafter, serially diluted inhibitors were added (20 μL/well), followed by the addition of HL2/3 cells (7.5 × 10^4^/well). After incubation for 6 h at 37 °C in 5% CO_2_, fusion efficiency was determined by measuring the luciferase activity using the Luciferase Assay System (Promega, Madison, WI) on a SpectraMax M5 plate reader (Molecular Devices, Sunnyvale, CA).

### HIV-1 infection assay

The antiviral activities of chimeric N-peptides and T20 against infection by HIV-1_IIIB_, HIV-1_BaL_, and T20-resistant HIV-1 strains were determined as previously described^13^. Briefly, 1 × 10^4^ MT-2 cells were infected with an HIV-1 strain (100 times the half-maximal tissue culture infective dose) in 200 μL of culture medium with or without the serially diluted test compounds overnight. Assay plates were incubated for 72 h, after which 100 μL of culture supernatant was collected from each well and assayed for p24 antigen using ELISA as described previously. The EC_50_ value of each test compound was calculated using Calcusyn software^33^,^38^.

### Cytotoxicity assay

The cytotoxic effect of the compounds on MT-2 cells was determined using the XTT assay as described previously^13^. Briefly, 100 μL of chimeric N-peptides and T20 at graded concentrations was added to 100 μL of uninfected MT-2 cells (5 × 10^5^/mL) prepared and incubated identically to that described above for the viral infection assay but measuring cell viability by adding 50 μL of XTT solution (1 mg/mL) with 0.02 μM phenazine methosulfate (PMS).

### CD spectroscopy

All measurements were performed on an MOS-450 system (BioLogic, Claix, France) at a 4.0 nm bandwidth, 0.1 nm resolution, 0.1 cm path length, 4.0 s response time and 50 nm/min scanning speed. The individual N- and C-peptides as well as their equimolar mixtures were prepared in PBS (pH 7.4) at a final concentration of 10 μM and incubated at 37 °C for 30 min. Thermal denaturation was monitored by the ellipticity change at 222 nm by application of a thermal gradient from 15 °C to 90 °C with a 2 °C interval.

### Sedimentation velocity analysis (SVA)

SVA was performed using a ProteomelabTMXL-A/XL-I analytical ultracentrifuge (Beckman Coulter, Fullerton, CA) equipped with a three-channel cell in an An-60 Ti rotor, as described previously^20^. All samples were prepared at final concentration of 45 μM in PBS and were incubated at 37 °C for 30 min and were initially scanned at 3000 rpm for 10 min. Data were collected at 60,000 rpm at a wavelength of 280 nm at 20 °C. Weight-averaged molecular weights were calculated by fitting each data file individually using the SEDFIT program.

### Native-PAGE

Equimolar mixtures of C34 and N-peptides (80 μM in PBS, pH 7.2) were incubated at 37 °C for 30 min. After mixing with 1: 1 Tris-glycine native sample buffer (BioRad, Hercules, CA), the individual N- and C- peptides and the mixture (40 μM in PBS, pH 7.2) were loaded (25 μL in each well) onto 12% Tris-glycine gels. Gel electrophoresis was run under a constant voltage of 120 V at room temperature for 2.5 h. The gel was then stained with Coomassie blue R250.

### Metabolic stability

Three male SD rats weighing approximately 200 g each were obtained from the Animal Center of Beijing Institute of Pharmacology & Toxicology and were used for the metabolic stability assay. Animals were treated in accordance with the Animal Welfare Act and the “Guide for the Care and Use of Laboratory Animals” (NIH Publication 86-23, revised 1985). ***Preparation of Samples.***Calibration samples were prepared by mixing various concentrations of each test compound ranging from 4 to 150 μM in H_2_O with rat plasma or tissue homogenate at a volume ratio of 100 μL:100 μL (Supplementary Figs S5–S7). Each test sample (100 μL) was spiked with 100 μL of acetonitrile containing 0.1% TFA. The mixture was vortexed and centrifuged at 18,000 *g* for 10 min. Then, the supernatant was transferred to HPLC autosampler vials, and aliquots (10 μL) were injected into the HPLC system. ***HPLC analysis.***HPLC analysis of (IZ14N24N)_3_ and (ccIZ14N24N)_3_ in rat plasma and tissue homogenates was performed on an LC-10AT VP Plus liquid chromatography (Shimadzu Corporation, Kyoto, Japan) with an SPD-10A VP Plus UV-Vis detector operating at 210 nm and quantified using SHIMADZU LCsolution Lite. The mobile phase was composed of solvent A (water containing 0.1% TFA) and solvent B (70% acetonitrile and 0.1% TFA). For (IZ14N24N)_3_, the HPLC separation was performed by a gradient method of solvent B from 10% to 45% over 5 min, from 45% to 80% over 15 min, from 80% to 100% over 2 min, and holding the column at 100% B for 2 min; whereas for (ccIZ14N24N)_3_, a gradient method of solvent B from 10% to 65% over 5 min, from 65% to 80% over 14 min, and from 80% to 90% over 5 min was used. The HPLC analysis was performed on a Waters X-Bridge C8 column (4.6 × 250 mm, 5 μm, 300 Å) at ambient temperature upon injection of 20 μL of each standard and/or sample to obtain the chromatogram. The only exception was for (IZ14N24N)_3_ in plasma and kidney homogenate, which was analyzed using a Phenomenex Jupiter C4 column (4.6 × 250 mm, 5 μm, 300 Å).

## Additional Information

**How to cite this article**: Wang, C. *et al*. Site-specific Isopeptide Bridge Tethering of Chimeric gp41 N-terminal Heptad Repeat Helical Trimers for the Treatment of HIV-1 Infection. *Sci. Rep*. **6**, 32161; doi: 10.1038/srep32161 (2016).

## Supplementary Material

Supplementary Information

## Figures and Tables

**Table 1:** Fusion and viral inhibitory activity and selectivity indexes of compounds.

Compd	EC_50_ (nM) for cell-cell fusion	EC_50_ (nM) for inhibition		CC_50_ (μM)^a^	SI^b^
		HIV-1_IIIB_ replication	HIV-1_BaL_ replication		
(IZN17L)_3_	20.2 ± 0.81	1.6 ± 0.1	4.5 ± 1.1	7.9 ± 1.3	4938
(IZN17M)_3_	13.9 ± 3.4	1.6 ± 0.1	4.0 ± 0.9	6.0 ± 0.5	3750
(IZN17R)_3_	10.7 ± 2.1	0.4 ± 0.3	3.8 ± 0.9	7.8 ± 0.9	19500
(IZ17N17)_3_	46.0 ± 4.6	4.1 ± 3.1	9.0 ± 0.2	4.0 ± 0.3	976
(IZ14N17)_3_	21.9 ± 2.4	1.9 ± 1.2	3.3 ± 2.0	7.3 ± 1.7	3842
(IZ10N17)_3_	190 ± 4.6	59.4 ± 9.1	74.7 ± 6.0	2.0 ± 0.2	34
(IZ14N24N)_3_	2.79 ± 0.1	0.4 ± 0.01	3.2 ± 0.9	>20	>50000
(IZ10N24N)_3_	28.3 ± 4.3	1.8 ± 0.7	4.7 ± 0.5	6.9 ± 1.0	3833
(IZ14N24C)_3_	6.40 ± 1.6	4.0 ± 2.3	6.6 ± 0.2	7.7 ± 0.8	1925
(IZ10N24C)_3_	13.8 ± 1.5	4.4 ± 0.4	11.4 ± 5.5	2.0 ± 0.4	454
(CCIZN17)_3_	13.8 ± 9.8	1.0 ± 0.2	4.4 ± 1.8	>20	>20000
T20	3.38 ± 1.1	4.9 ± 0.6	25.2 ± 1.9	>20	>4081

The EC_50_ data were derived from the results of three independent experiments and expressed as means ± standard deviation.

^a^CC_50_ = half maximal cytotoxic concentration.

^b^SI (selectivity index) = CC_50_/EC_50_ for inhibiting HIV-1_IIIB_ infection.

**Table 2:** The activity of (IZ14N24N)_3_ against infection by T20-resistant variants.

NL4-3 mutant	T20 (EC_50_, nM)	(IZ14N24N)_3_ (EC_50_, nM)
D36G^a^	18.9 ± 2.0	1.3 ± 0.2
(36G)V38A^b^	250 ± 33 (13)	0.7 ± 0.1 (0.5)
(36G)V38A/N42D^b^	150 ± 28 (8)	2.7 ± 0.1 (2)
(36G)N42T/N43K^b^	720 ± 84 (38)	3.3 ± 0.1 (2.5)
(36G)V38E/N42S^b^	1427 ± 118 (76)	1.2 ± 0.2 (0.9)
(36G)V38A/N42T^b^	961 ± 53 (51)	1.3 ± 0.8 (1)

Values represent the mean ± standard deviation of three independent experiments; the values in parentheses denote the relative changes (n-fold) to the EC_50_ of the T20-sensitive strain.

^a^T20-sensitive strain.

^b^T20-resistant strains.
