# Supplementary material for: Site-specific Isopeptide Bridge Tethering of Chimeric gp41 N-terminal Heptad Repeat Helical Trimers for the Treatment of HIV-1 Infection
Source: Sci Rep. 2016 Aug 26;6:32161. doi: 10.1038/srep32161 (PMC4999862; doi:10.1038/srep32161)
Supplement: Supplementary Information [file srep32161-s1.pdf]

Supplementary Information

Site-specific Isopeptide Bridge Tethering of Chimeric gp41 N-terminal Heptad Repeat Helical Trimers for the Treatment of HIV-1 Infection

Chao Wang,[1,†] Xue Li,[1,†] Fei Yu,[2] Lu Lu,[2] Xifeng Jiang,[1] Xiaoyu Xu,[1] Huixin Wang,[4] Wenqing Lai,[1] Tianhong Zhang,[1] Zhenqing Zhang,[1] Ling Ye,[5] Shibo Jiang[2,3,*] and Keliang Liu[1,*]

[1]State Key Laboratory of Toxicology and Medical Countermeasures, Beijing Institute of Pharmacology & Toxicology, 27 Tai-Ping Road, Beijing, 100850, China;
[2]Key Laboratory of Medical Molecular Virology of Ministries of Education and Health, School of Basic Medical Sciences, Fudan University, Shanghai 200032, China;
[3]Lindsley F. Kimball Research Institute, New York Blood Center, New York, NY 10065, USA;
[4]School of Pharmaceutical Engineering, Shenyang Pharmaceutical University, Shenyang, 110016, China;
[5]Department of Microbiology and Immunology and Emory Vaccine Center, Emory University School of Medicine, Atlanta, GA 30322, USA.

[†]These authors contributed equally to this work.

Corresponding Authors:

K.L.: State Key Laboratory of Toxicology and Medical Countermeasures, Beijing Institute of Pharmacology & Toxicology, 27 Tai-Ping Road, Beijing, 100850, China; Tel.: 86-10-6816-9363; Fax: 86-10-6821-1656, E-mail: keliangliu55@126.com.

S.J.: Key Laboratory of Medical Molecular Virology of Ministries of Education and Health, Shanghai Medical College and Institute of Medical Microbiology, Fudan University, Shanghai 200032, China; Tel.: 86-21-54237673; Fax: 86-21-54237465; E-mail: shibojiang@fudan.edu.cn.

Supplementary Table S1. Designed peptides to assess the specificity of acyl transfer reaction. X highlighted in bold represents Glu with side chain thioester.

Entry	Compound	Sequence
A1	IZ(SBn)N17L	IKK**X** IEAI**K**KE QEAIKKK IEAIEKL LQLTVWG IKQLQAR IL
A2	IZ(SBn)N17LR	IKK**X** IEAI**R**KE QEAIKKK IEAIEKL LQLTVWG IKQLQAR IL
B1	IZ(SBn)N17M	IKKE IEAIKK**X** QEAI**K**KK IEAIEKL LQLTVWG IKQLQAR IL
B2	IZ(SBn)N17MR	IKKE IEAIKK**X** QEAI**R**KK IEAIEKL LQLTVWG IKQLQAR IL
C1	IZ(SBn)N17R	IKKE IEAIKKE QEAIKK**K** IEAI**X**KL LQLTVWG IKQLQAR IL
C2	IZ(SBn)N17RR	IKKE IEAIKKE QEAIKK**R** IEAI**X**KL LQLTVWG IKQLQAR IL
D1	IZ17(SBn)N17	IKKE QEAIKK**K** IEAI**X**KL LQLTVWG IKQLQAR IL
D2	IZ17(SBn)N17R	IKKE QEAIKK**R** IEAI**X**KL LQLTVWG IKQLQAR IL
E1	IZ14(SBn)N17	E QEAIKK**K** IEAI**X**KL LQLTVWG IKQLQAR IL
E2	IZ14(SBn)N17R	E QEAIKK**R** IEAI**X**KL LQLTVWG IKQLQAR IL
F1	IZ10(SBn)N17	IKK**K** IEAI**X**KL LQLTVWG IKQLQAR IL
F2	IZ10(SBn)N17R	IKK**R** IEAI**X**KL LQLTVWG IKQLQAR IL
G1	IZ14(SBn)N24N	E QEAIKK**K** IEAI**X**KA IEAQQHL LQLTVWG IKQLQAR IL
G2	IZ14(SBn)N24NR	E QEAIKK**R** IEAI**X**KA IEAQQHL LQLTVWG IKQLQAR IL
H1	IZ14(SBn)N24C	E QEAIKK**K** IEAI**X**KL LQLTVWG IKQLQAR ILAVERY LK
H2	IZ14(SBn)N24CR	E QEAIKK**R** IEAI**X**KL LQLTVWG IKQLQAR ILAVERY LK
I1	IZ10(SBn)N24N	IKK**K** IEAI**X**KA IEAQQHL LQLTVWG IKQLQAR IL
I2	IZ10(SBn)N24NR	IKK**R** IEAI**X**KA IEAQQHL LQLTVWG IKQLQAR IL
J1	IZ10(SBn)N24C	IKK**K** IEAI**X**KL LQLTVWG IKQLQAR ILAVERY LK
J2	IZ10(SBn)N24CR	IKK**R** IEAI**X**KL LQLTVWG IKQLQAR ILAVERY LK

Supplementary Table S2. Biophysical properties of the designed peptides. [a] CD spectra of each designed chimeric N-peptide was monitored in PBS, pH 7.4. The final concentration of the peptide was 10 μM. [b] Helicity (%) of the 6HBs formed between chimeric N-peptides and C34 calculated on the basis of the CD spectra using θ_{222nm} of 33000 for 100 % helicity.

Compd	Helicity (%) [a]	*Tm* (℃) [a]	Helicity (%) of chimeric N-peptide/C34 complexes[b]
$(IZN17L)_3$	96	>90	74
$(IZN17M)_3$	100	>90	86
$(IZN17R)_3$	100	>90	80
$(IZ17N17)_3$	98	>90	70
$(IZ14N17)_3$	100	>90	74
$(IZ10N17)_3$	75	>90	73
$(IZ14N24N)_3$	100	>90	87
$(IZ14N24C)_3$	100	>90	76
$(IZ10N24N)_3$	100	>90	80
$(IZ10N24C)_3$	100	>90	63

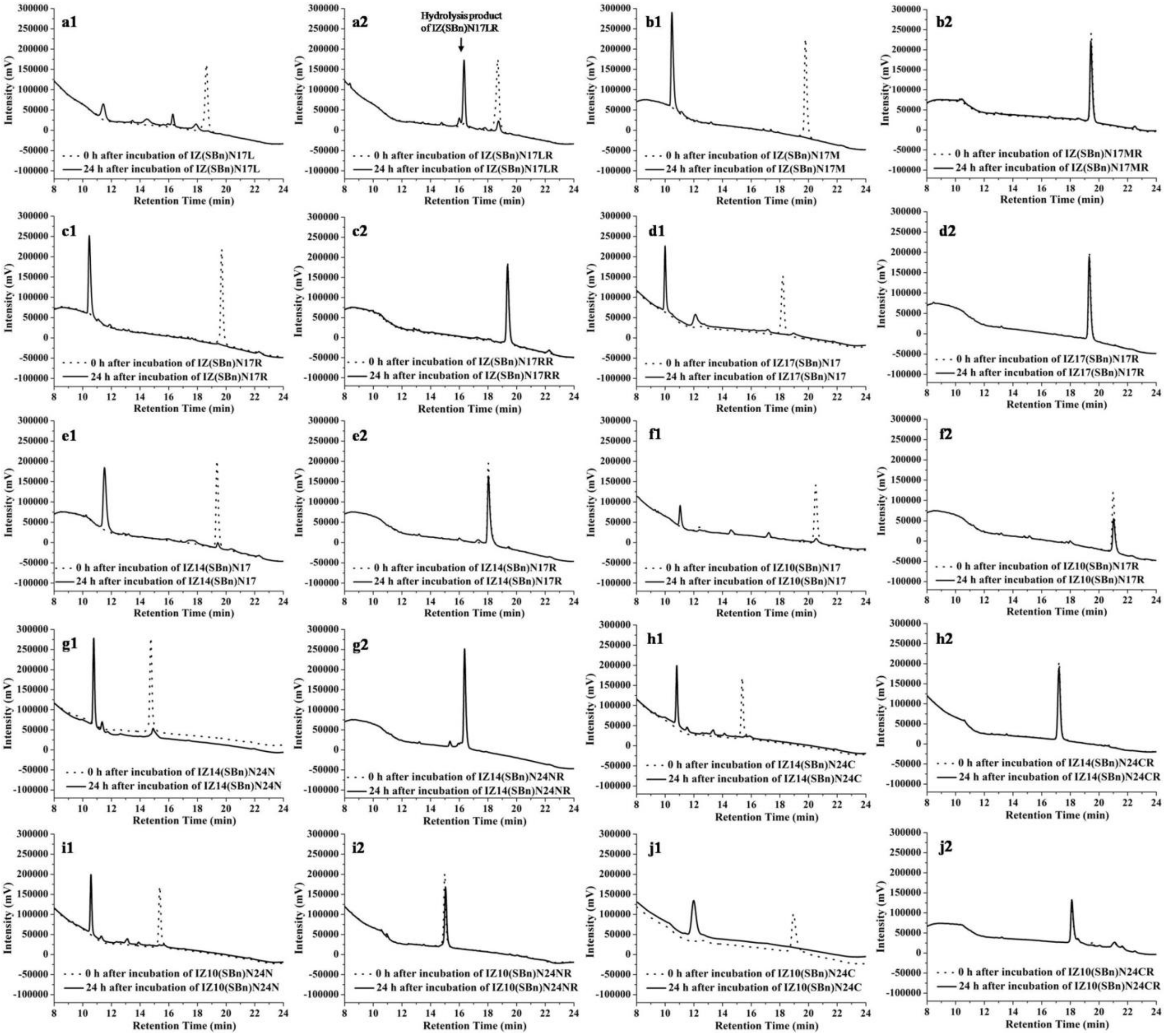

Supplementary Figure S1. The specificity of the interhelical acyl-transfer reaction. (**a1-j1**) RP-HPLC traces for Lys-Glu ligation of thioester intermediates at t=0 and 24 h. (**a2-j2**) RP-HPLC traces of control peptides in which the reactive Lys residues were mutated to Arg at t=0 and 24 h.

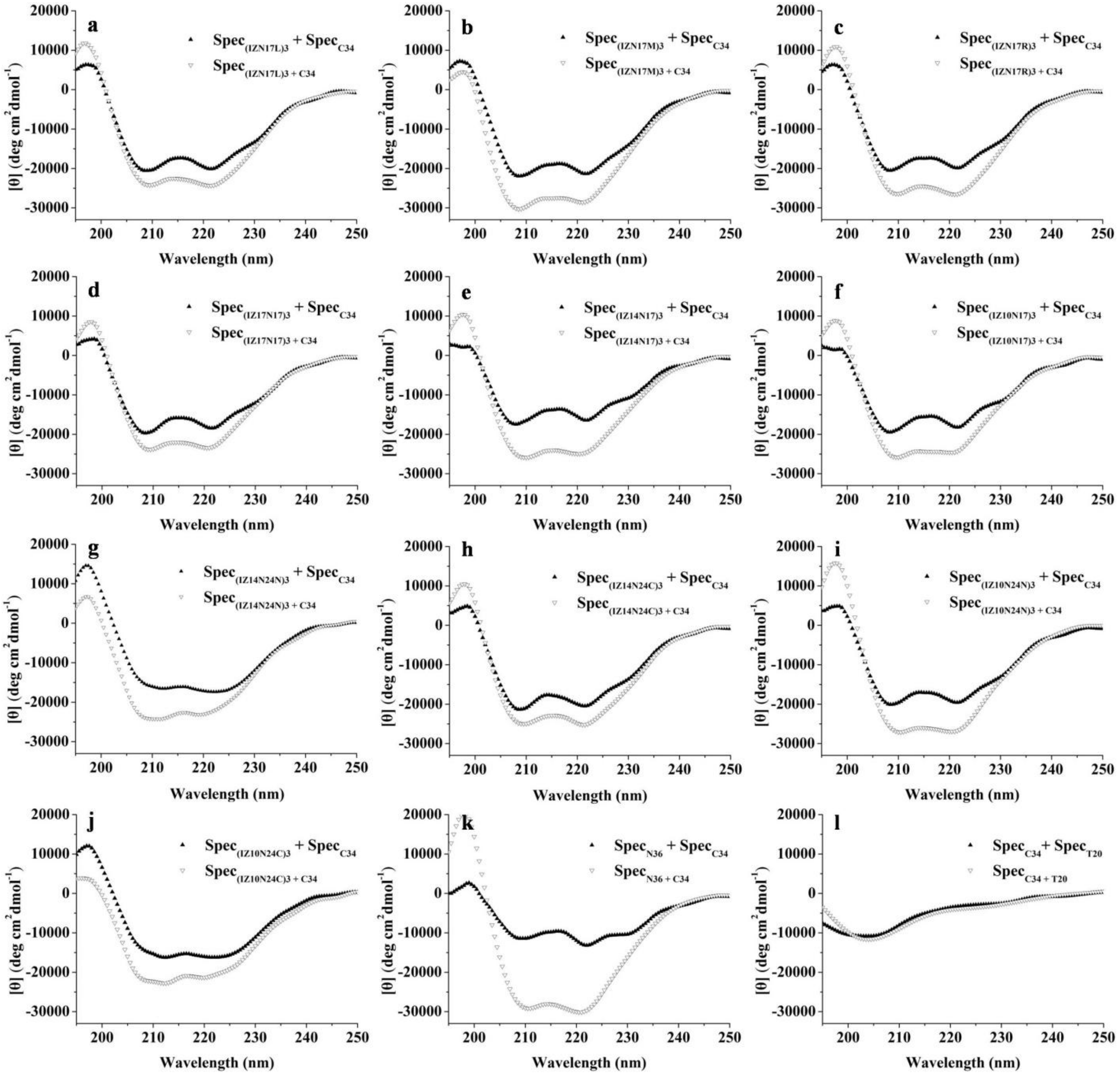

Supplementary Figure S2. C34 and covalently stabilized N-trimers show interaction in solution. CD spectrum of peptide mixtures ($Spec_{N+C}$, ▽) and the sum of the spectra of the related isolated peptides ($Spec_N$ + $Spec_C$, ▲) are shown for comparison. The covalently stabilized N-trimers-C34 interaction induces more α-helix structure than the sum of the single peptides. Final concentration of each peptide in PBS is 10 μM.

Supplementary Figure S3. Analytical HPLC of designed N-peptides. Compound purity was determined by analytical HPLC on a reversed-phase C8 column (Zorbax Eclipse XDB-C8, 5 µm, 4.6 mm × 150 mm) using two different solvent systems. Method A: a gradient method increasing linearly from 10% to 50% solvent B over 5 min and then from 50% to 100% solvent B over 15 min, and decreasing linearly to 5% solvent B over 3 min, at a flow rate of 1 mL/min. Method B: a gradient method increasing linearly from 10% to 40% solvent B over 5 min and then from 50% to 90% solvent B over 15 min, and decreasing linearly to 10% solvent B over 3 min, at a flow rate of 1 mL/min. Compounds were detected by UV absorption at 210 nm with a Shimadzu SPD-10A detector.

Analytical HPLC of $(IZN17L)_3$
[99 % purity; t_R (Method A) = 10.1 min]

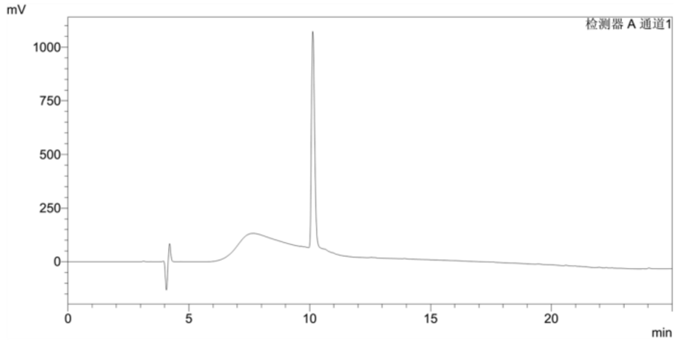

Analytical HPLC of $(IZN17L)_3$
[98 % purity; t_R (Method B) = 15.8 min]

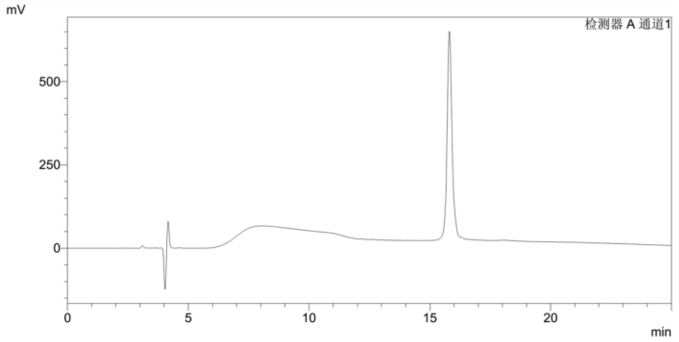

Analytical HPLC of $(IZN17M)_3$
[99 % purity; t_R (Method A) = 10.0 min]

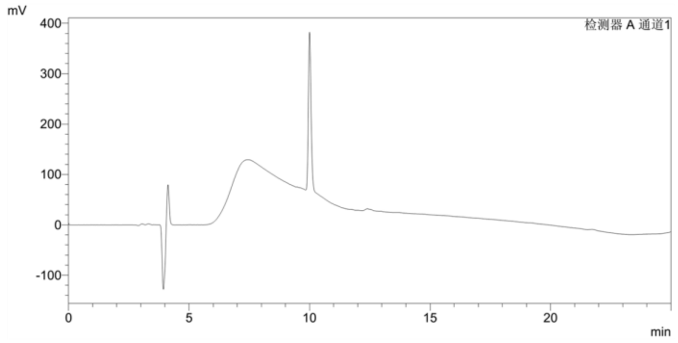

Analytical HPLC of $(IZN17M)_3$
[96 % purity; t_R (Method B) = 15.8 min]

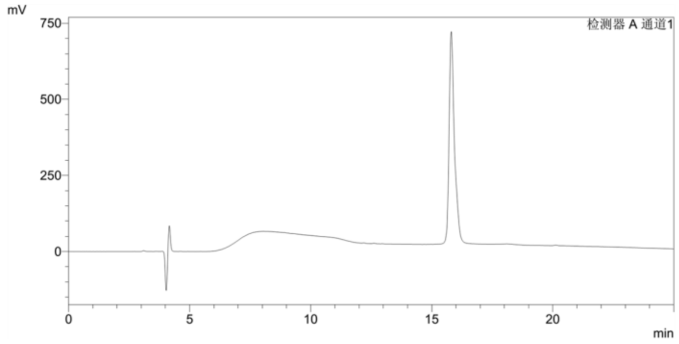

Analytical HPLC of $(IZN17R)_3$
[99 % purity; t_R (Method A) = 10.0 min]

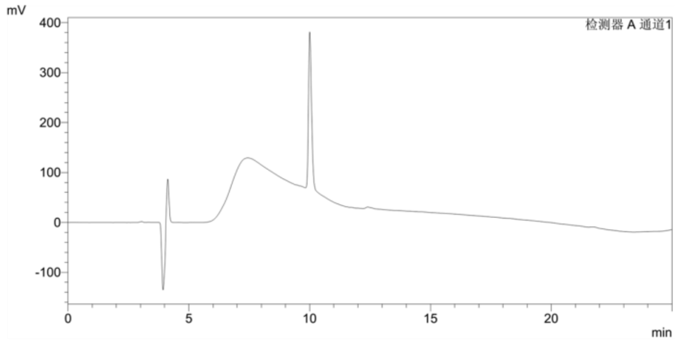

Analytical HPLC of $(IZN17R)_3$
[96% purity; t_R (Method B) = 15.7 min]

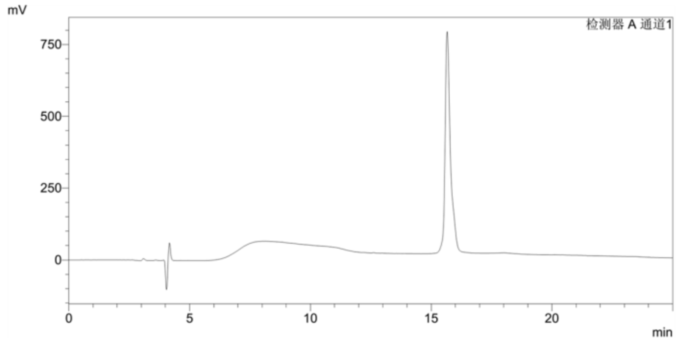

Analytical HPLC of (IZ17N17)$_3$
[99% purity; t_R (Method A) = 10.0min]

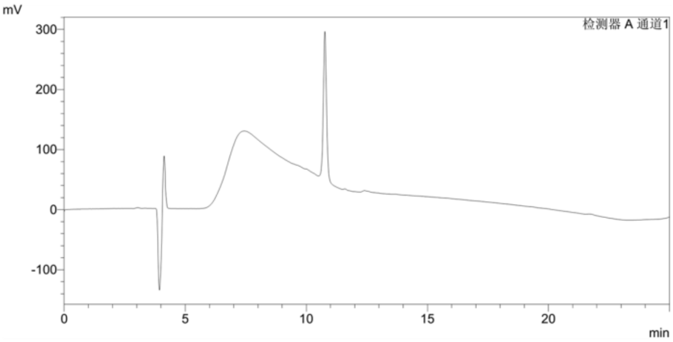

Analytical HPLC of (IZ17N17)$_3$
[99% purity; t_R (Method B) = 16.1 min]

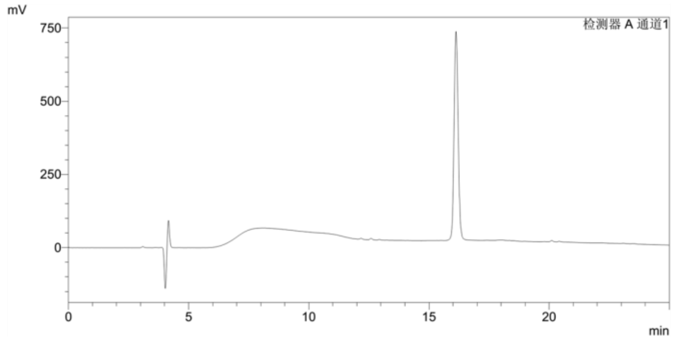

Analytical HPLC of (IZ14N17)$_3$
[99% purity; t_R (Method A) = 10.9min]

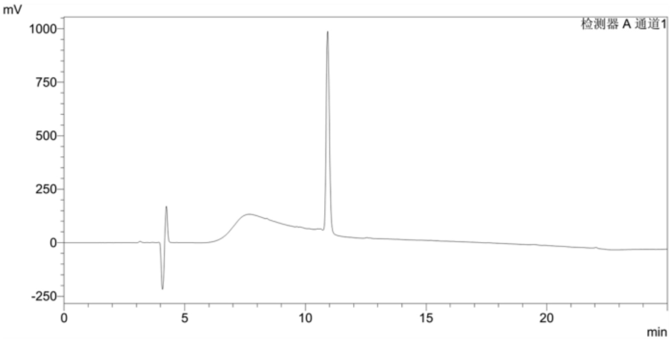

Analytical HPLC of (IZ14N17)$_3$
[97% purity; t_R (Method B) = 20.4min]

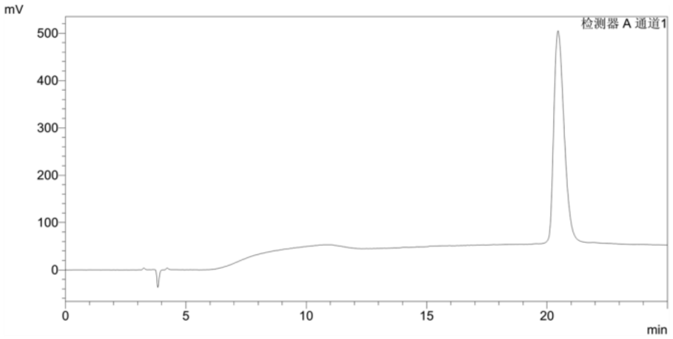

Analytical HPLC of (IZ10N17)$_3$
[99% purity; t_R (Method A) = 11.1min]

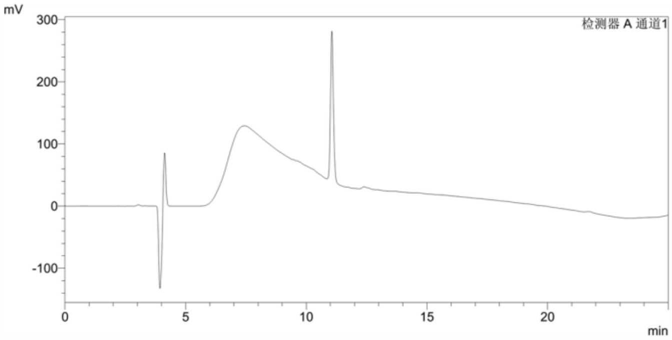

Analytical HPLC of (IZ10N17)$_3$
[98% purity; t_R (Method B) = 20.6min]

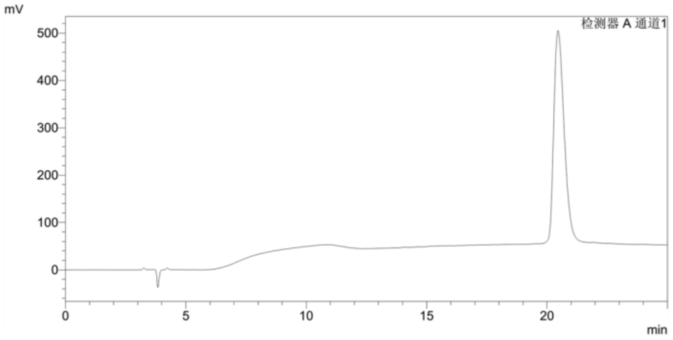

Analytical HPLC of (IZ14N24N)$_3$
[99% purity; t_R (Method A) = 10.6min]

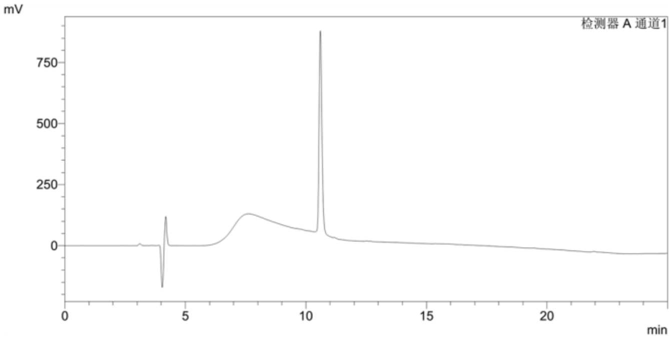

Analytical HPLC of (IZ14N24N)$_3$
[99% purity; t_R (Method B) = 18.0min]

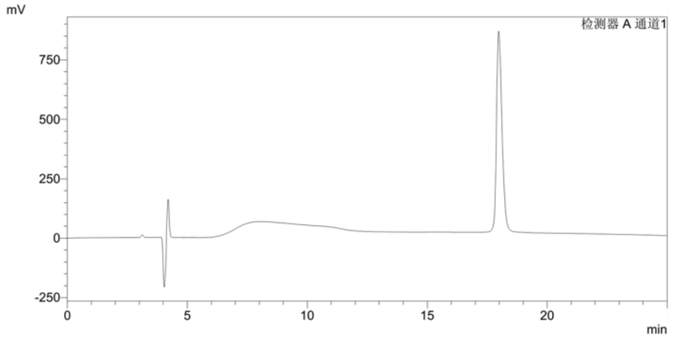

Analytical HPLC of (IZ10N24N)$_3$
[99% purity; t_R (Method A) = 10.7min]

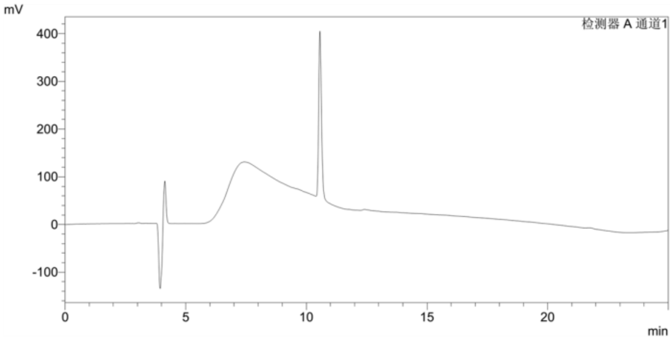

Analytical HPLC of (IZ10N24N)$_3$
[97% purity; t_R (Method B) = 19.2min]

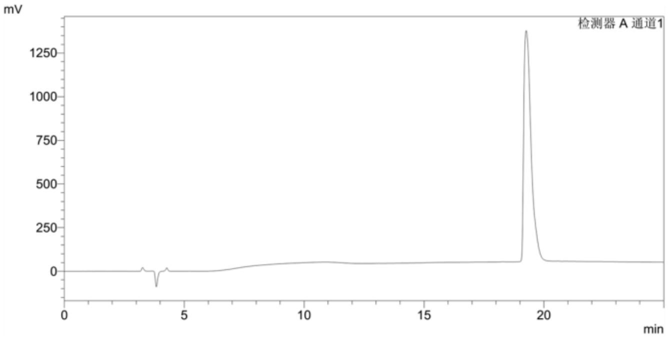

Analytical HPLC of (IZ14N24C)$_3$
[99% purity; t_R (Method A) = 10.6min]

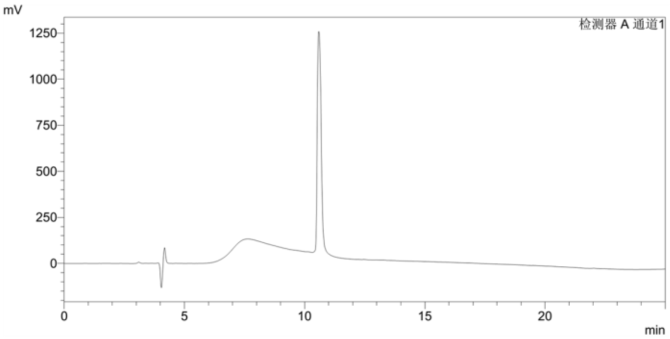

Analytical HPLC of (IZ14N24N)$_3$
[99% purity; t_R (Method B) = 18.0min]

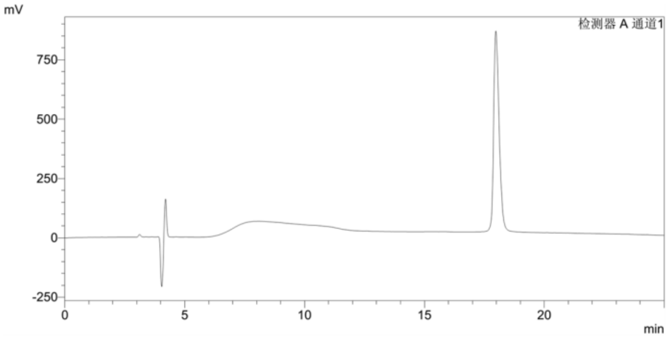

Analytical HPLC of (IZ10N24C)$_3$
[98% purity; t_R (Method A) = 11.7min]

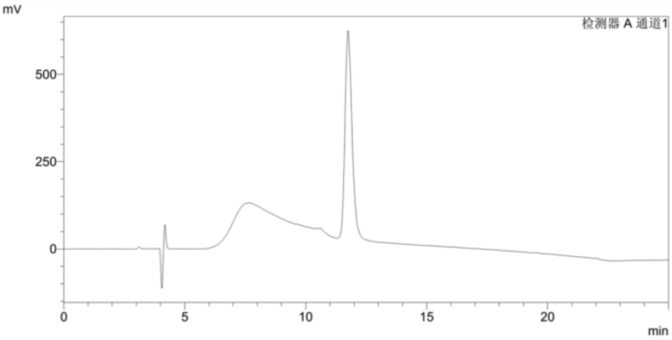

Analytical HPLC of (IZ10N24C)$_3$
[98% purity; t_R (Method B) = 21.7min]

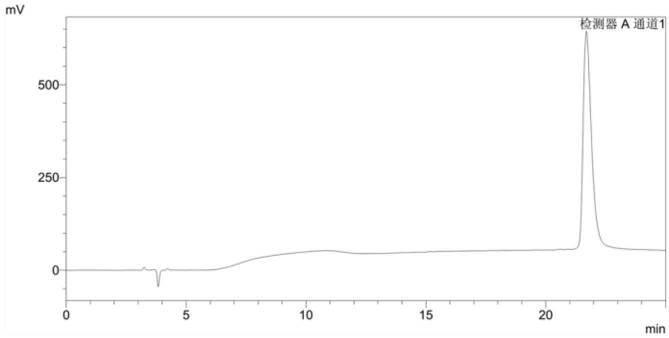

Analytical HPLC of (CCIZN17)$_3$
[98% purity; t_R (Method A) = 15.4min]

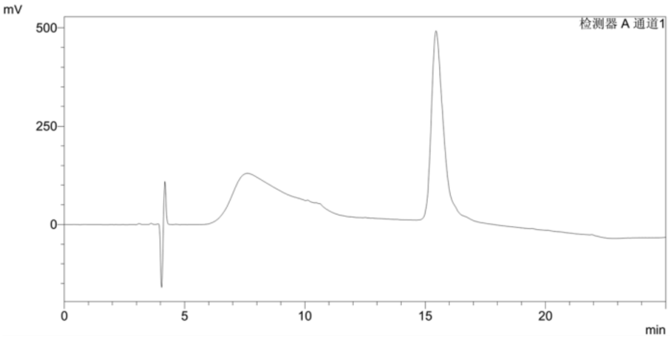

Analytical HPLC of (CCIZN17)$_3$
[96% purity; t_R (Method B) = 26.7min]

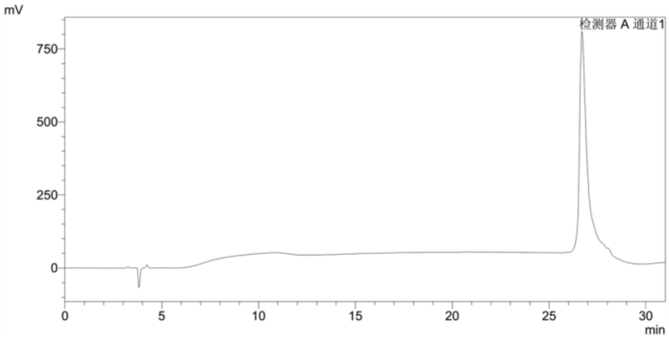

Supplementary Figure S4. MALDI-TOF-MS of designed N-peptides.

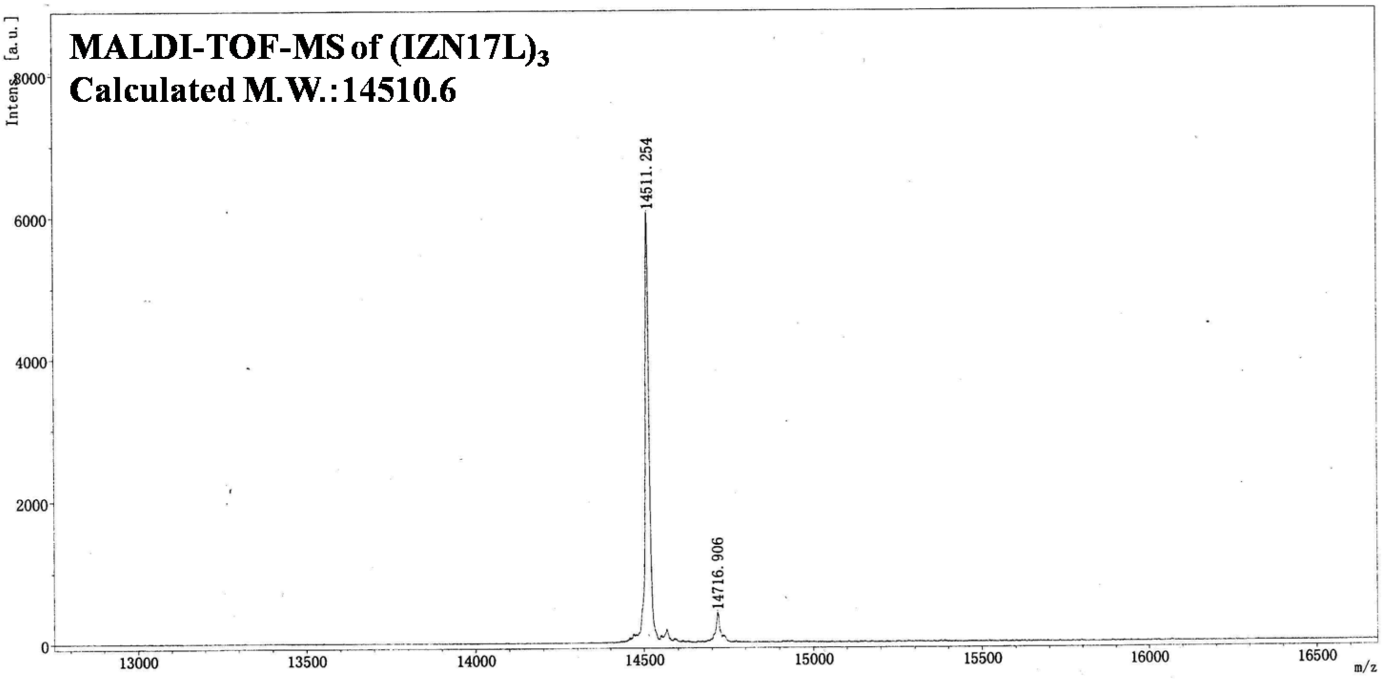

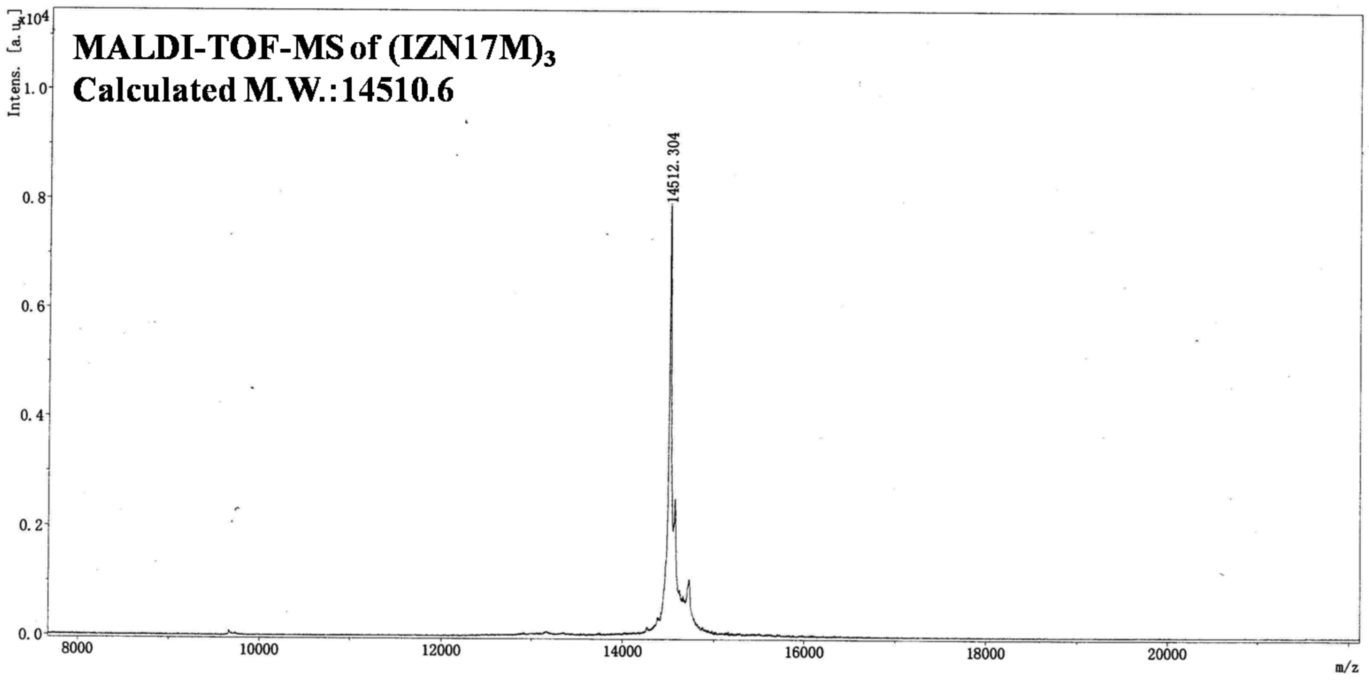

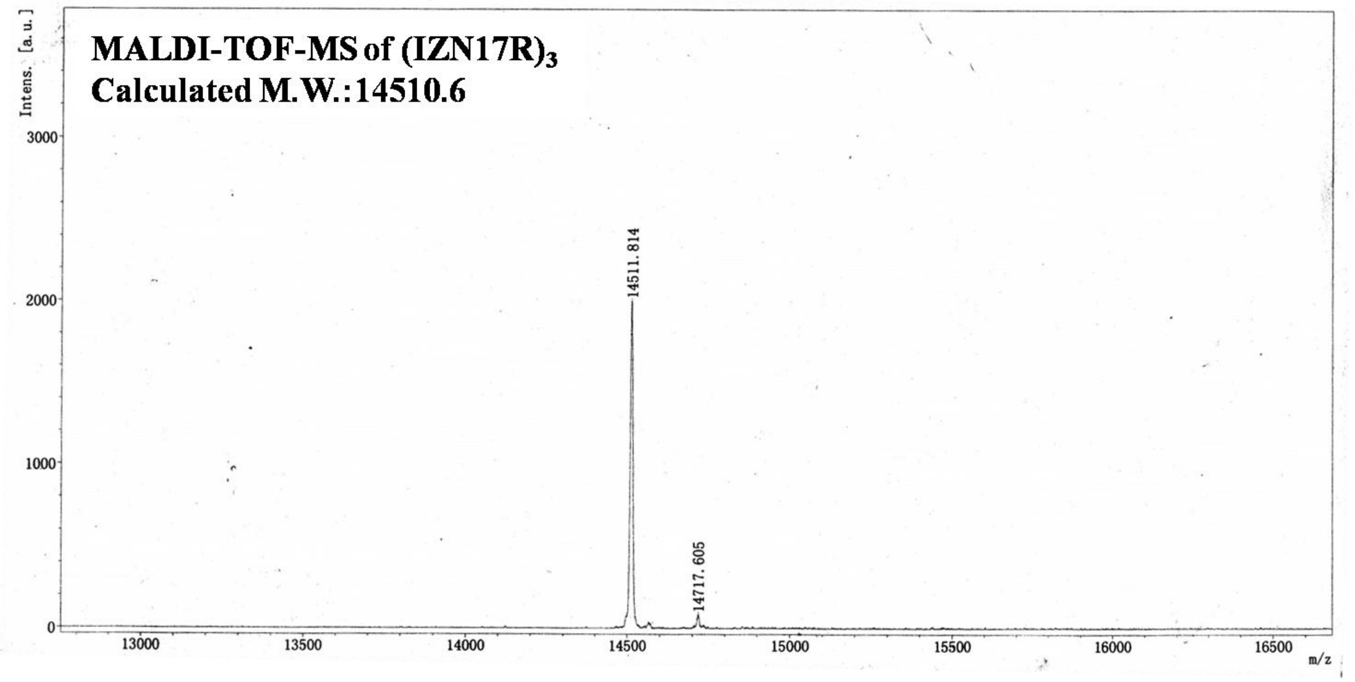
MALDI-TOF-MS of $(IZN17R)_3$
Calculated M.W.:14510.6
Intens. [a. u.]
14511.814
14717.605
m/z

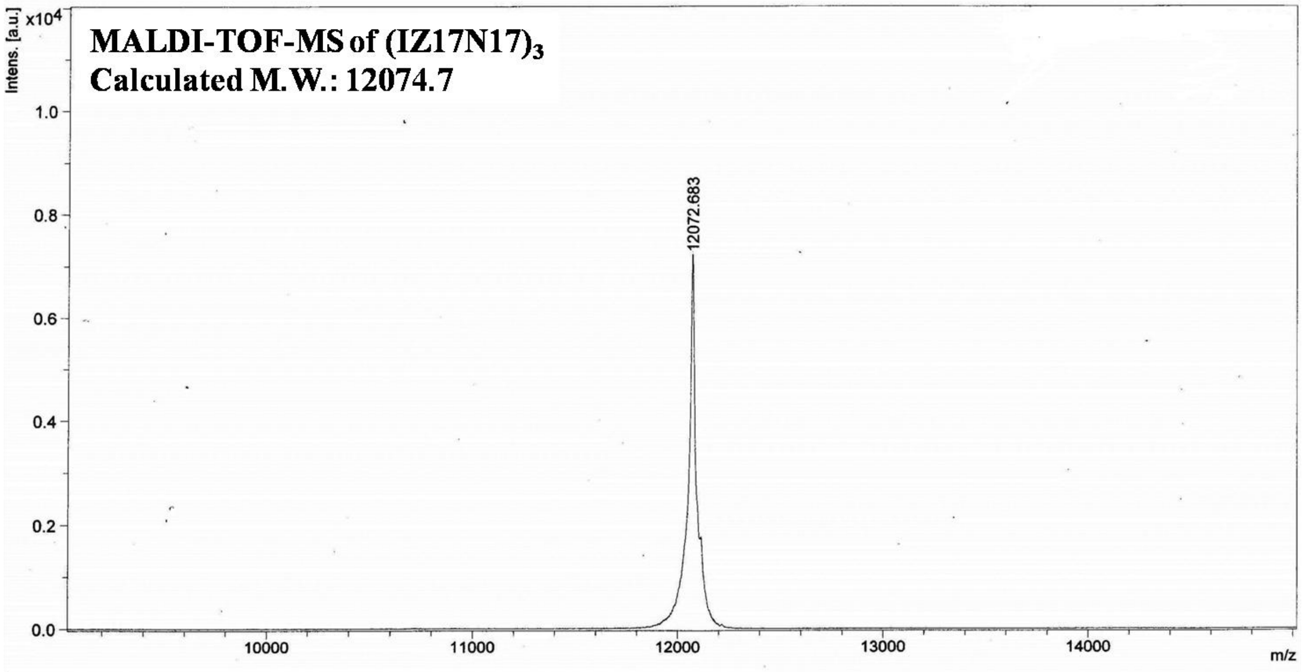
MALDI-TOF-MS of $(IZ17N17)_3$
Calculated M.W.: 12074.7
Intens. [a.u.]
x10^4
12072.683
m/z

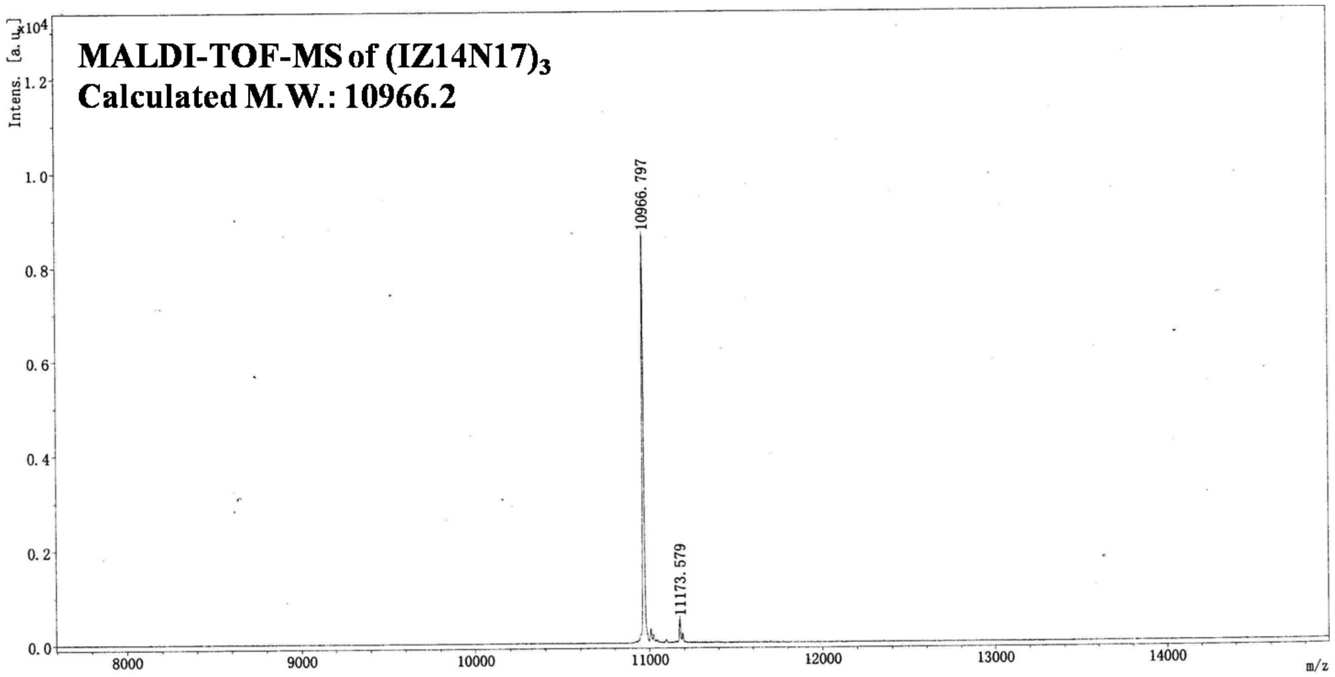
MALDI-TOF-MS of $(IZ14N17)_3$
Calculated M.W.: 10966.2
Intens. [a. u.]
x10^4
10966.797
11173.579
m/z

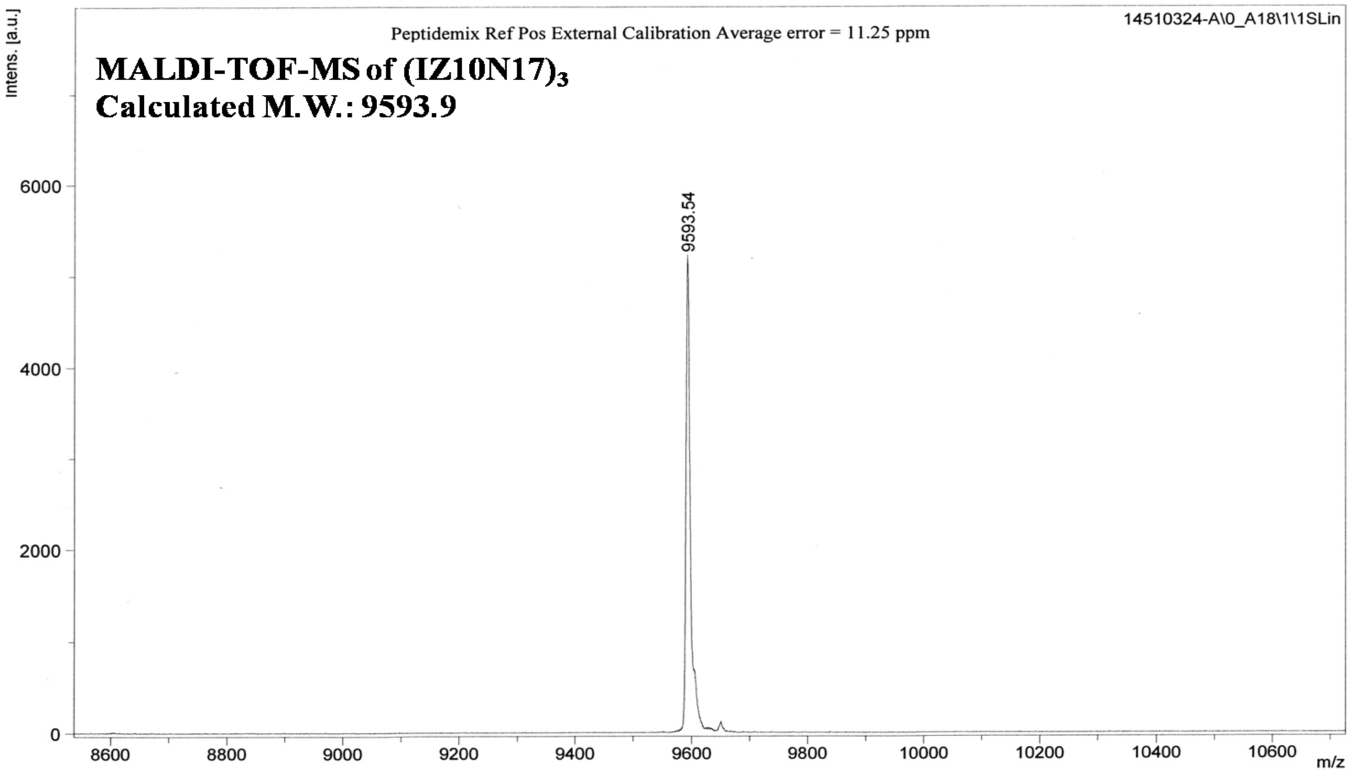
Peptidemix Ref Pos External Calibration Average error = 11.25 ppm
14510324-A\0_A18\1\1SLin
MALDI-TOF-MS of $(IZ10N17)_3$
Calculated M.W.: 9593.9
Intens. [a.u.]
9593.54
m/z

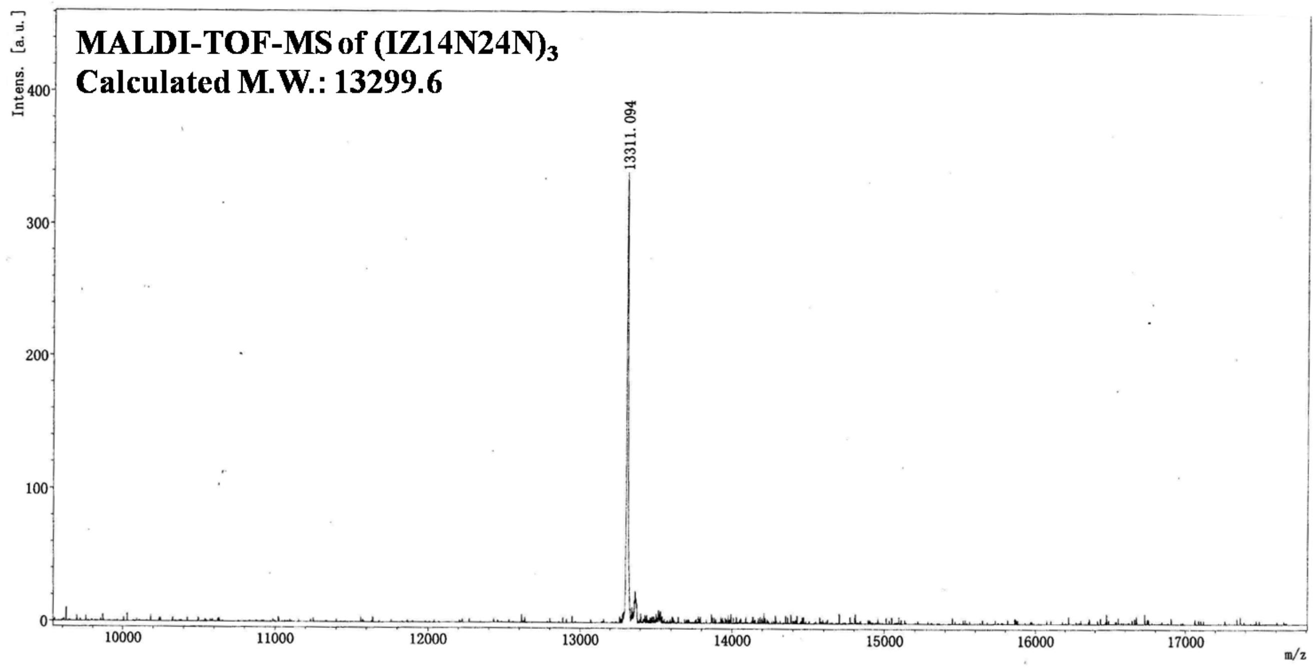
MALDI-TOF-MS of $(IZ14N24N)_3$
Calculated M.W.: 13299.6
13311.094
Intens. [a. u.]
m/z

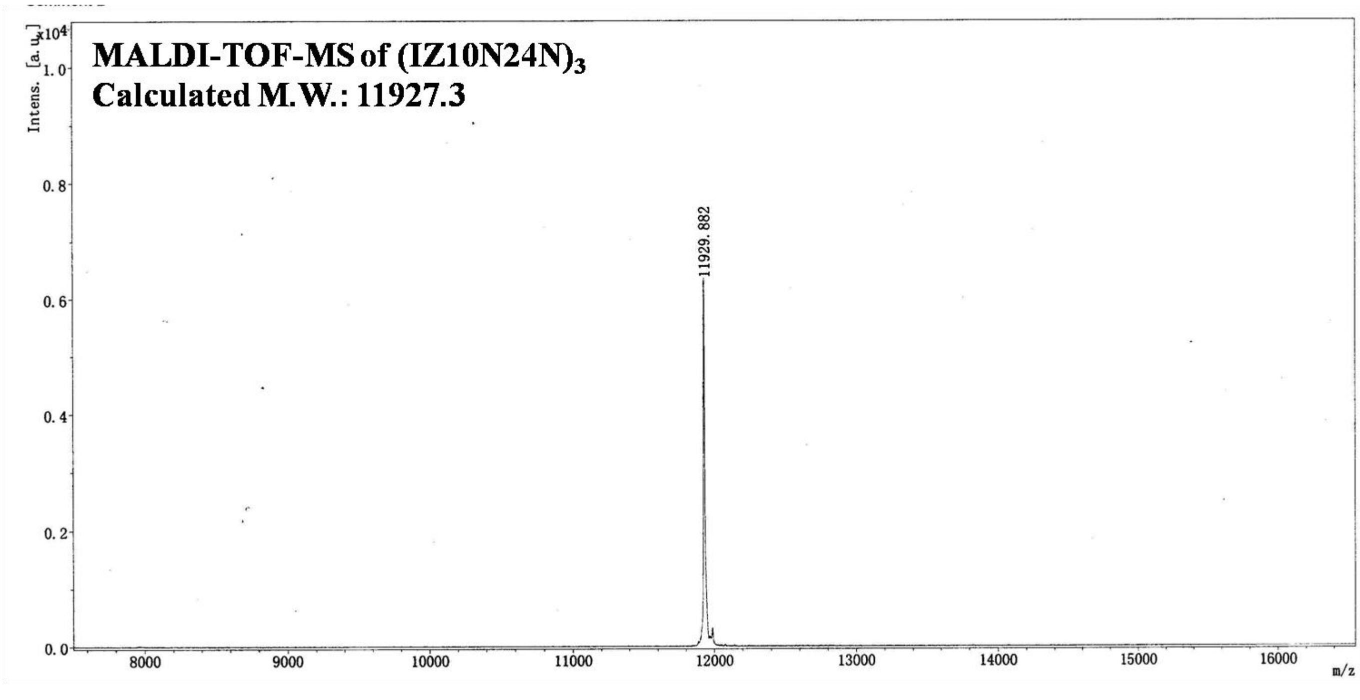
MALDI-TOF-MS of $(IZ10N24N)_3$
Calculated M.W.: 11927.3
11929.882
Intens. [a. u.]
x10^4
m/z

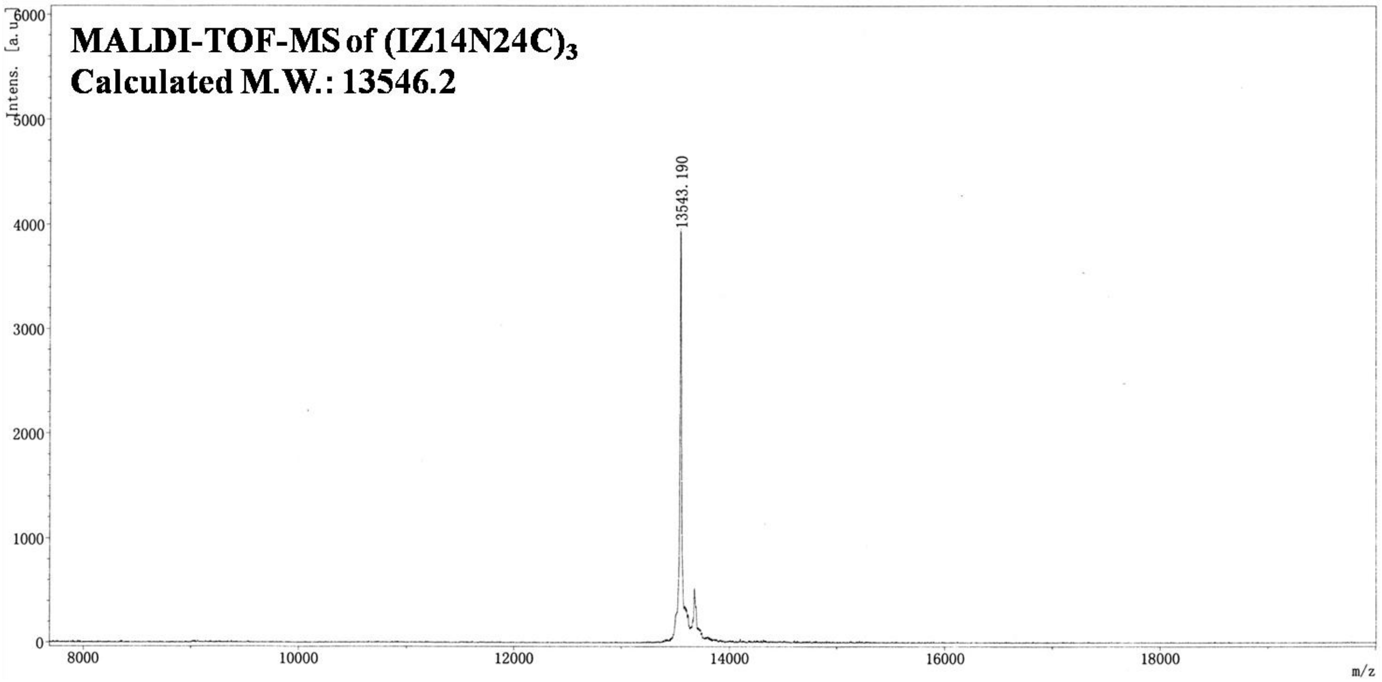
MALDI-TOF-MS of (IZ14N24C)3
Calculated M.W.: 13546.2
Intens. [a.u.]
13543.190
m/z

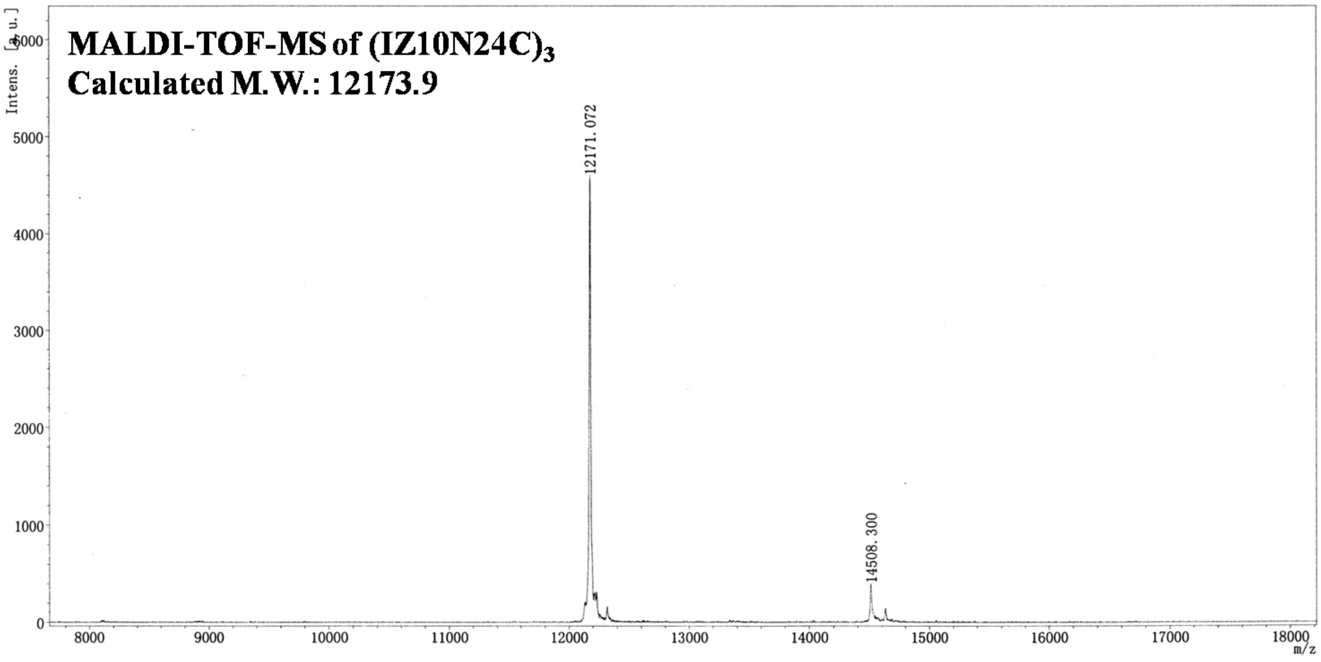
MALDI-TOF-MS of (IZ10N24C)3
Calculated M.W.: 12173.9
Intens. [a.u.]
12171.072
14508.300
m/z

Comment 2

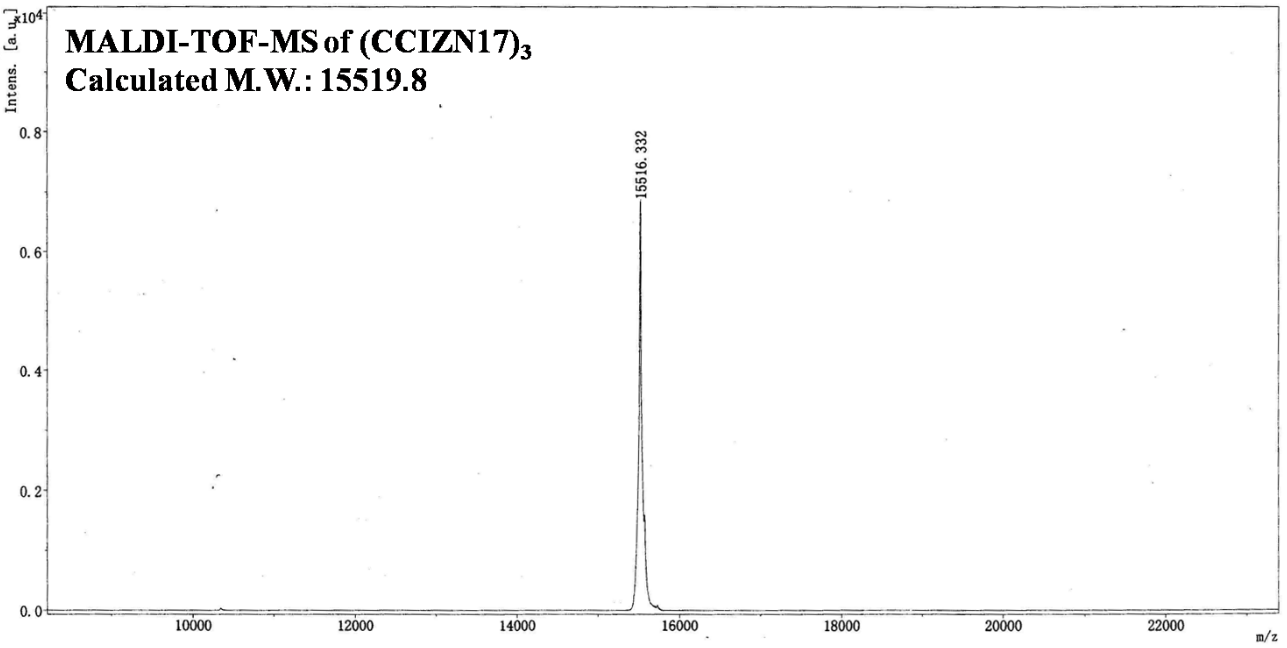

MALDI-TOF-MS of (CCIZN17)3
Calculated M.W.: 15519.8
15516.332
Intens. [a. u.]
x10^4
m/z

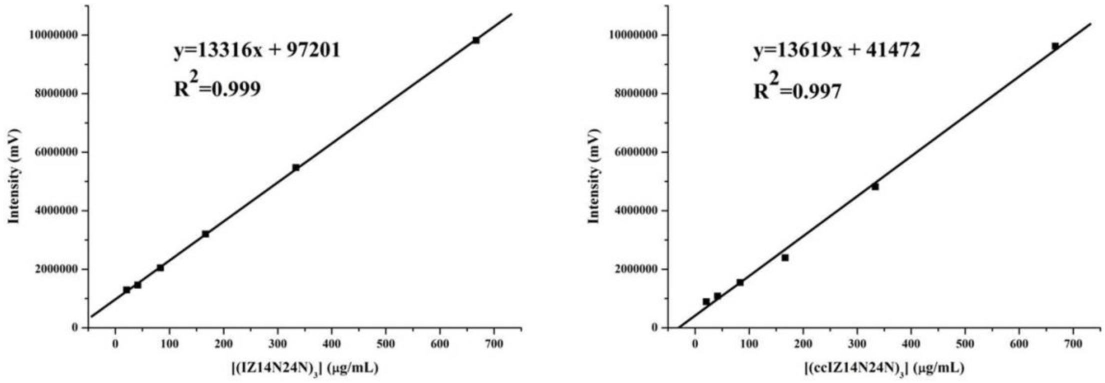

Supplementary Figure S5. Calibration curves used to quantitate $(IZ14N24N)_3$ and $(ccIZ14N24N)_3$ in rat plasma.

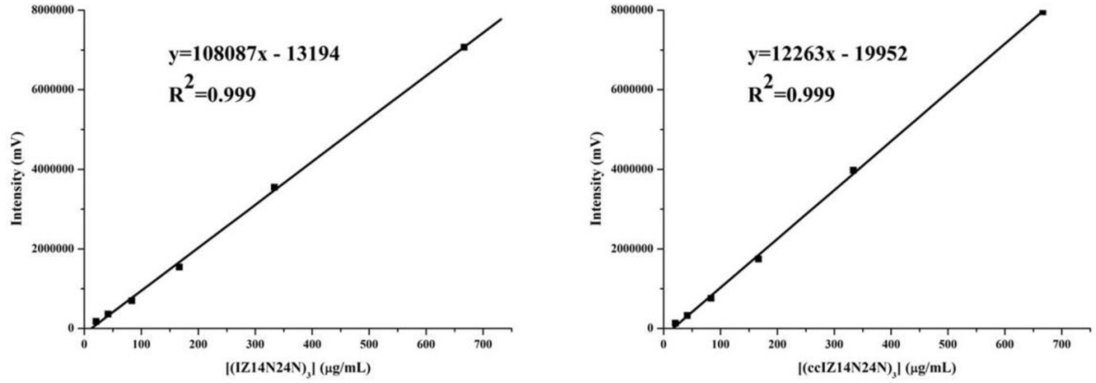

Supplementary Figure S6. Calibration curves used to quantitate $(IZ14N24N)_3$ and $(ccIZ14N24N)_3$ in liver homogenates.

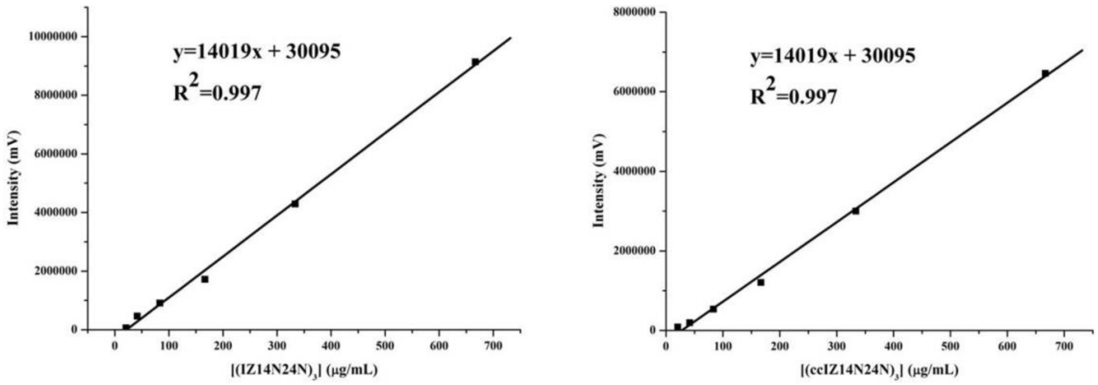

Supplementary Figure S7. Calibration curves used to quantitate $(IZ14N24N)_3$ and $(ccIZ14N24N)_3$ in kidney homogenates.
